# Galectin-3 as a Marker and Potential Therapeutic Target in Breast Cancer

**DOI:** 10.1371/journal.pone.0103482

**Published:** 2014-09-25

**Authors:** Hao Zhang, Xi Liang, Chao Duan, Caigang Liu, Zuowei Zhao

**Affiliations:** 1 Department of Breast Surgery, the Second Hospital of Dalian Medical University, Dalian, China; 2 Department of Cardiothoracic, Benxi Central Hospital, Benxi, China; National University of Ireland Galway, Ireland

## Abstract

Galectin-3 has a relatively high level of expression in triple-negative breast cancers and is a potential marker for this disease. However, the clinical and prognostic implications of galectin-3 expression in breast cancer remain unclear. We examined mastectomy specimens from 1086 breast cancer cases and matching, adjacent non-cancerous tissues using immunohistochemistry. Overall, triple-negative breast cancers expressed galectin-3 more strongly than did other breast cancers types (63.59% vs 21.36%, P = 0.001). Galectin-3 expression was not found to be an independent prognostic factor for breast cancer by Cox regression analysis, but was associated with chemotherapeutic resistance. Apoptosis was only weakly induced by arsenic trioxide (ATO) treatment in galectin-3-positive breast cancer cells (MDA-MB-231 and MCF-7), although ATO treatment up-regulated galectin-3 expression. Knockdown of galectin-3 in MDA-MB-231 cells sensitized them to killing by ATO. These findings support a possible role for galectin-3 as a marker for triple-negative breast cancer progression and as a therapeutic target in combination with ATO treatment, although the mechanisms that underlie this synergy require further investigation.

## Introduction

Breast cancer is the most lethal of the female-specific malignancies [1]. Over the last 30 years, deaths due to breast cancer have approximately tripled in Japan, which historically has had a low incidence of this disease, and globally, the World Health Organization estimates that more than 1.2 million people are diagnosed with breast cancer each year [2].

Breast cancer is a heterogeneous disease encompassing several different phenotypes with different biological characteristics [3]. Preventive agents and targeted therapies directed at estrogen receptor, progesterone receptor, and human epidermal growth factor 2 receptor (HER2/neu) have resulted in improved clinical outcomes for many women with breast cancer, but formidable challenges remain in treating tumors that do not express these molecular targets. These “triple-negative” breast carcinomas (TNCs) represent 15% of newly diagnosed breast cancer cases and often exhibit a basal epithelial or basal-like gene expression profile that is associated with poor survival [4]. Development of new therapeutic agents for these clinically intractable tumors is an important goal. Previous studies have shown that arsenic trioxide (ATO) can inhibit cell growth and induce apoptosis in a variety of human cancers, including breast cancer [5], [6]. However, the molecular mechanisms that underlie this inhibition remain largely unknown. A better understanding of the molecular and physiological properties of ATO could help to establish its therapeutic potential in breast cancer.

Galectin-3 is a 30 kD protein of the non-integrin β-galactoside-binding lectin family 29 and has a role in cell adhesion, cell migration, cell growth, cell cycle regulation, apoptosis, and the cellular repair process [8]–[11]. It has also been shown to be expressed at higher levels in TNC compared to non-TNC tumors [7], and to prevent nitric oxide-induced apoptosis in breast cancer cells [12], suggesting that it has a role in breast cancer development and progression. However, the clinical and prognostic significance of galectin-3 expression in breast cancer remain unclear.

To date there have been no reported studies that address galectin-3 expression in the breast tumors of Asian patients. Therefore, we examined 1086 mastectomy specimens obtained from breast cancer patients in order to investigate the expression of galectin-3 in relation to clinicopathological features, survival, and chemotherapeutic resistance. We also evaluated the role of galectin-3 in the ATO-induced apoptosis of breast cancer cell lines using shRNA-mediated knockdown.

## Materials and Methods

### Patients and tissue specimens

A total of 1187 patients who had histologically confirmed breast cancer and who underwent radical resection in the Tumor Hospital of Liaoning Province, China Medical University, and Dalian Medical University between January 2001 and January 2007 were enrolled into this study. The inclusion criteria were (1) performance of a curative operation, (2) pathological examination of resected specimens, (3) pathological examination of more than 10 lymph nodes after the operation, and (4) availability of a complete medical record including the ER, PR, Her2, p53, and Ki-67 status. The study protocol was approved by the Ethics Committee of Dalian Medical University, China Medical University and Liaoning Tumor Hospital (the ethics approval numbers were 201301007, 6234557, and 2013121342, respectively), and written informed consent was obtained from all participants involved in the study.

### Immunohistochemistry

Thin slices of tumor tissue (including breast cancer tissue, paracancerous tissue, and atypical hyperplasia tissue) from each patient were fixed in 4% formaldehyde solution (pH 7.0) for not more than 24 h. The tissues were processed routinely for paraffin embedding, and 4-µm-thick sections were cut and placed on glass slides coated with 3-aminopropyl triethoxysilane for immunohistochemistry. Tissue samples were stained with hematoxylin and eosin to determine the histological type and tumor grade.

Cells that were immunopositive for galectin-3 (1∶50, ab118851, Abcam, Cambridge, USA) appeared blown, with staining in the nucleus and/or cytoplasm. Staining was scored semi-quantitatively according to the following criteria: 0 if <1% of neoplastic cells discretely expressed galectin-3; 1+ if ≥1 and <10% of morphologically unequivocal neoplastic cells discretely expressed galectin-3; and 2+ if ≥10% of morphologically unequivocal neoplastic cells discretely expressed galectin-3. Samples scored as 1+ or 2+ were considered positive. The IHC scoring was independently determined by 3 pathologists.

### Measurement of cell viability

The inhibitory effects of ATO on breast carcinoma cells were determined using the 3-[4,5-dimethylthiazol-2-yl]-2,5-diphenyltetrazolium bromide (MTT) assay. Briefly, 3×10^3^ MDA-MB-231 or MCF-7 cells were plated in 96-well plates and allowed to attach overnight. Cells were treated with 0, 0.25, 0.5, 1, 2.5, 5, or 10 µM ATO (Harbin Yida Pharmaceutical Co., Ltd.) for 72 h at 37°C in 5% CO_2_. Each treatment was repeated for 5 independent wells. Twenty microliters of MTT solution (Sigma) solution (5 mg/ml in phosphate-buffered saline [PBS]) was added to each well, and the plates were incubated for an additional 4 h at 37°C. In order to solubilize the formazan crystal formed in viable cells, 150 µl dimethyl sulfoxide was added to each well before measuring the absorbance at 490 nm.

### Apoptosis assay

Apoptosis was measured using annexin V-fluorescein isothiocyanate (FITC) and propidium iodide (PI) double staining. Two hundred microliters of cells (1×10^6^ cells per ml) were centrifuged at 1000 rpm for 5 min (4°C). Cells were washed with pre-cooled PBS and re-suspended in 100 µl binding buffer with 2 µl annexin-V-FITC (20 µg/ml). The mixed samples were placed on ice in the dark for 15 min. Four hundred microliters of PBS and 1 µl PI (50 µg/ml) were added to each sample before measurement. The cells were then sorted and their fluorescence was measured 2 min later using a flow cytometer (Beckman Coulter EPICS XL). Cells without annexin V-FITC and PI staining were used as negative controls.

### Generation of stable galectin-3 knockdown cell lines

The cells were transfected with human Gal-3-specific (sc-35443) or control siRNAs (sc-37007; Santa Cruz Biotechnology, Dallas, Texas, USA) using Lipofectamine 2000 Reagent (Invitrogen, Carlsbad, CA, USA), according to the manufacturer’s protocol. Four candidate shRNAs were transduced into MDA-MB-231 cells. The target sequences were GR311: CCCACGCTTCAATGAGAACAA, GR312: GCAAACAGAATTGCTTTAGAT, and GR313: GCCACTGATTGTGCCTTATAA.

### Statistical analysis

All data were analyzed with SPSS statistics software (Version 17.0, Chicago, IL, USA). Relationships between tumor marker and other parameters were assessed using the χ^2^-test or Fisher’s exact test. Linear regression was used to study the correlation between various clinicopathological features and galectin-3 protein expression. Regression (r) values were regarded as indicating no correlation (0.0–0.2), or a low (0.2–0.4), moderate (0.4–0.6), marked (0.6–0.8), or high degree of correlation (0.8–1.0). Logistic regression was carried out to assess the factors related to post-operative distant metastasis. A P-value of less than 0.05 was considered statistically significant.

## Results

### Galectin-3 expression and clinicopathological features

**Figure 1:**
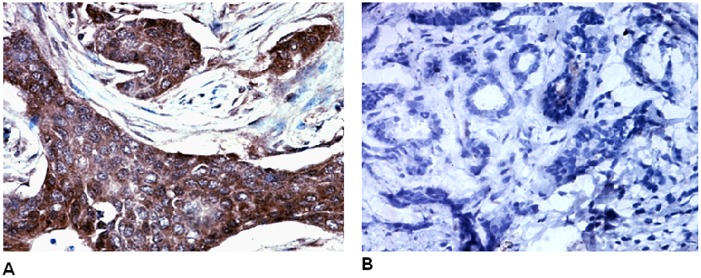
Immunohistochemical analysis revealed that galectin-3 was located in the cytoplasm and membrane of breast cancer cells (A). Galectin-3 protein is expressed at a significantly higher level in breast cancer tissues compared to paracancerous tissue (B).

Immunohistochemical examination revealed that galectin-3 was located in the cytoplasm and membrane of breast cancer cells, and was expressed significantly more strongly in breast tumors compared to paracancerous tissue (Figure 1).

In total, 388 (32.7%) of the 1187 breast cancer cases showed high galectin-3 expression, although the expression level varied significantly with respect to age, tumor size, histological grade, tumor stage, lymph node metastasis, and TNC (P = 0.023, 0.000, 0.000, 0.023, 0.001, and 0.000, respectively; Table 1). There were 251 cases of ductal carcinoma in situ (DCIS), 936 cases of invasive ductal carcinomas (IDC), and 618 cases with no lymph node metastasis. Overall, TNC tumors expressed galectin-3 more frequently than the other cancer types (57.96% vs. 26.74%; P = 0.000; Table 1).

**Table 1:** Galectin-3 expression and clinicopathological features (n = 1187).

Variables	Galectin-3^–^	Galectin-3^+^	*P* value
**Age**			0.023
<35 Y	139	89	
≥35 Y	660	299	
**Tumor size**			0.000
T1	156	47	
T2	557	318	
T3	76	17	
T4	10	6	
**Histological grade**			0.000
I	72	43	
II	694	78	
III	33	267	
**Tumor stage**			0.023
DCIS	154	97	
IDC	645	291	
**Metastatic nodes**			0.001
positive	356	213	
negative	443	175	
**Her-2 status**			0.251
positive	225	97	
negative	574	291	
**Triple-negative** **breast cancer**			0.000
yes	95	131	
no	704	257	

DCIS = ductal carcinoma in situ, IDC = invasive ductal carcinoma.

χ^2^-test was used to assess the relationships between tumor marker and other parameters.

P<0.05 was considered statistically significant.

### Galectin-3 expression and survival

Spearman correlation regression analysis revealed a linear relationship between galectin-3 expression and histological grade, lymph node metastasis, and TNC (r = 0.297, 0.371, and 0.403, respectively, P = 0.011, 0.001, and 0.000, respectively). A subsequent multivariate analysis revealed that histological grade, lymph node metastasis, and tumor size were all significantly associated with post-operative distant metastasis (P = 0.001, 0.003, and 0.001, respectively; Table 2). However, Cox regression analysis showed that galectin-3 expression was not an independent prognostic factor for breast cancer.

**Table 2:** Multivariate analysis of the factors related to post-operative distant metastasis.

Characteristic	Exp(B)	95% CI for Exp(B)	P value
Age	0.435	0.231–2.632	0.350
Tumor size	3.574	1.540–5.028	0.001
Histological grade	3.193	1.845–5.685	0.001
Tumor stage	2.464	0.739–4.219	0.067
Lymph node metastasis	3.476	1.031–5.514	0.003
Her-2 status	2.314	0.826–5.476	0.072
Galectin-3	2.611	0.573–9.585	0.057
Triple-negativebreast cancer	2.533	0.901–5.662	0.061

CI = confidence interval.

### Galectin-3 expression and chemotherapeutic resistance

We further studied the relationships between patient age, tumor size, histological grade, histological type, molecular type, cancer stem cell (CSC) ratio, galectin-3 expression, and chemotherapeutic sensitivity in 135 breast cancer cases involving neoadjuvant chemotherapy. This revealed that molecular type, CSC ratio, and galectin-3 expression were significantly associated with chemosensitivity (P = 0.007, 0.012, and 0.031, respectively). Galectin-3 was expressed in 20%, 21.7%, 43.8%, and 46.4% of tumors from complete response (CR), partial response (PR), stable disease (SD), and progressive disease (PD) patients, respectively. Correlations between Galectin-3 expression and chemotherapeutic resistance were significantly different among chemosensitivity (Table 3). According to each of the categories in Table 3, correlation between CR and PR, CR and SD, CR and PD, PR and SD, PR and PD, SD and PD were 0.535, 0.051, 0.033, 0.058, 0033, and 0.782, respectively.

**Table 3:** Correlations between Galectin-3 expression and chemotherapeutic resistance in breast cancers (n = 135).

Chemosensitivity	n	Galectin-3^–^	Galectin-3^+^	Galectin-3^+^ *%*	*P* value
					0.021
**CR**	15	12	3	20%	
**PR**	60	51	13	21.7%	
**SD**	32	18	14	43.8%	
**PD**	28	15	13	46.4%	

CR: complete response; PR: partial response; SD: stable disease; PD: progressive disease.

### ATO induces apoptosis in breast cancer cells

**Figure 2:**
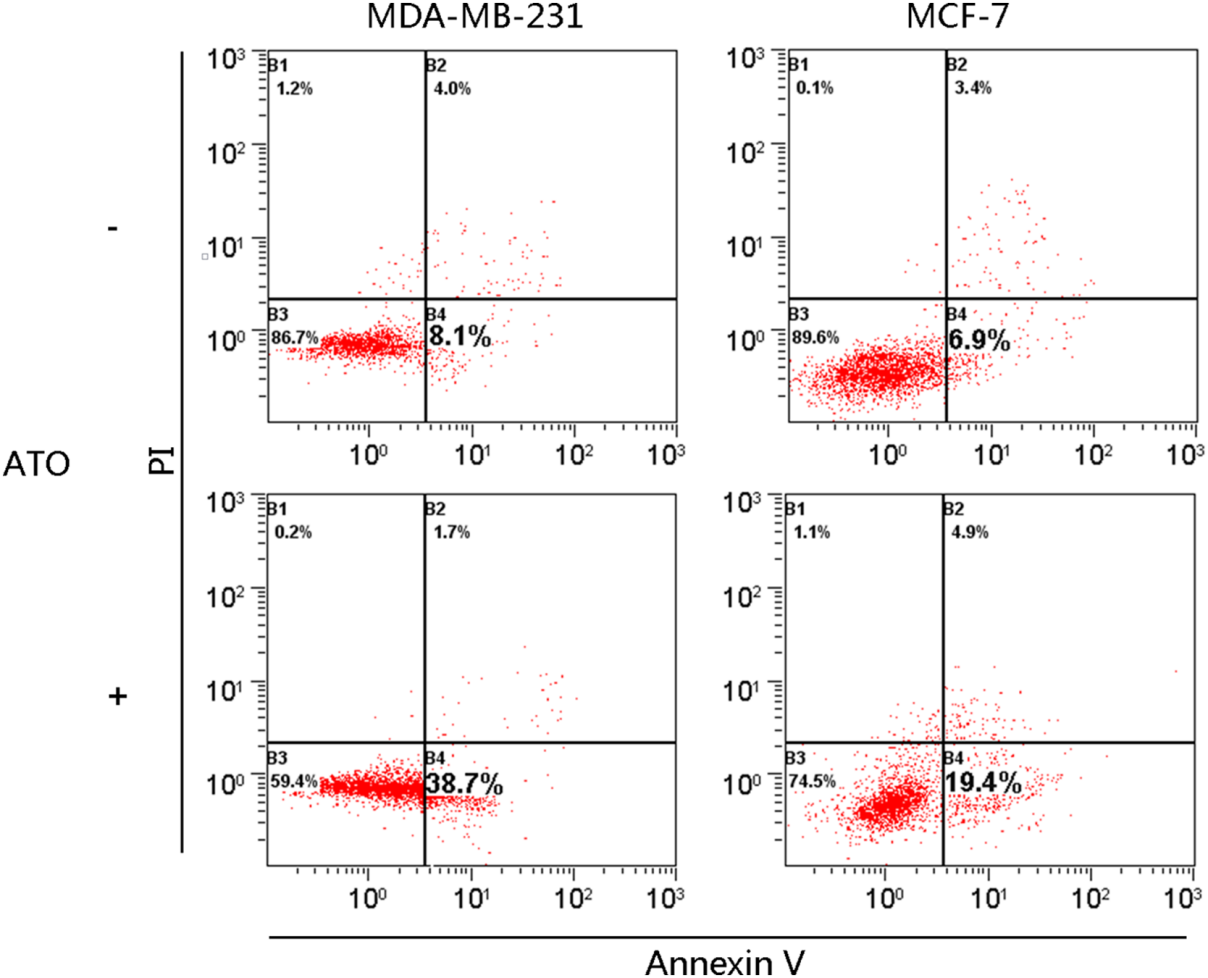
ATO treatment (2.5 µM) induces limited apoptosis in breast cancer cells. Untreated cells (top panel) and cells treated with ATO (bottom panel) were then analyzed by staining with PI and annexin V, followed by flow cytometry. The proportion of cells in apoptosis is shown in the figure.

We tested whether ATO induced apoptosis in breast cancer cell lines using an initial concentration range of 2.5–10 µM. We treated 2 breast cancer cell lines (MDA-MB-231 and MCF-7) with a concentration of 2.5 µM ATO for 72 h and compared the level of apoptosis using PI and annexin V-FITC double staining. ATO induced apoptosis in both MDA-MB-231 and MCF-7 cells (Figure 2).

### Galectin-3 expression before and after ATO treatment

**Figure 3:**
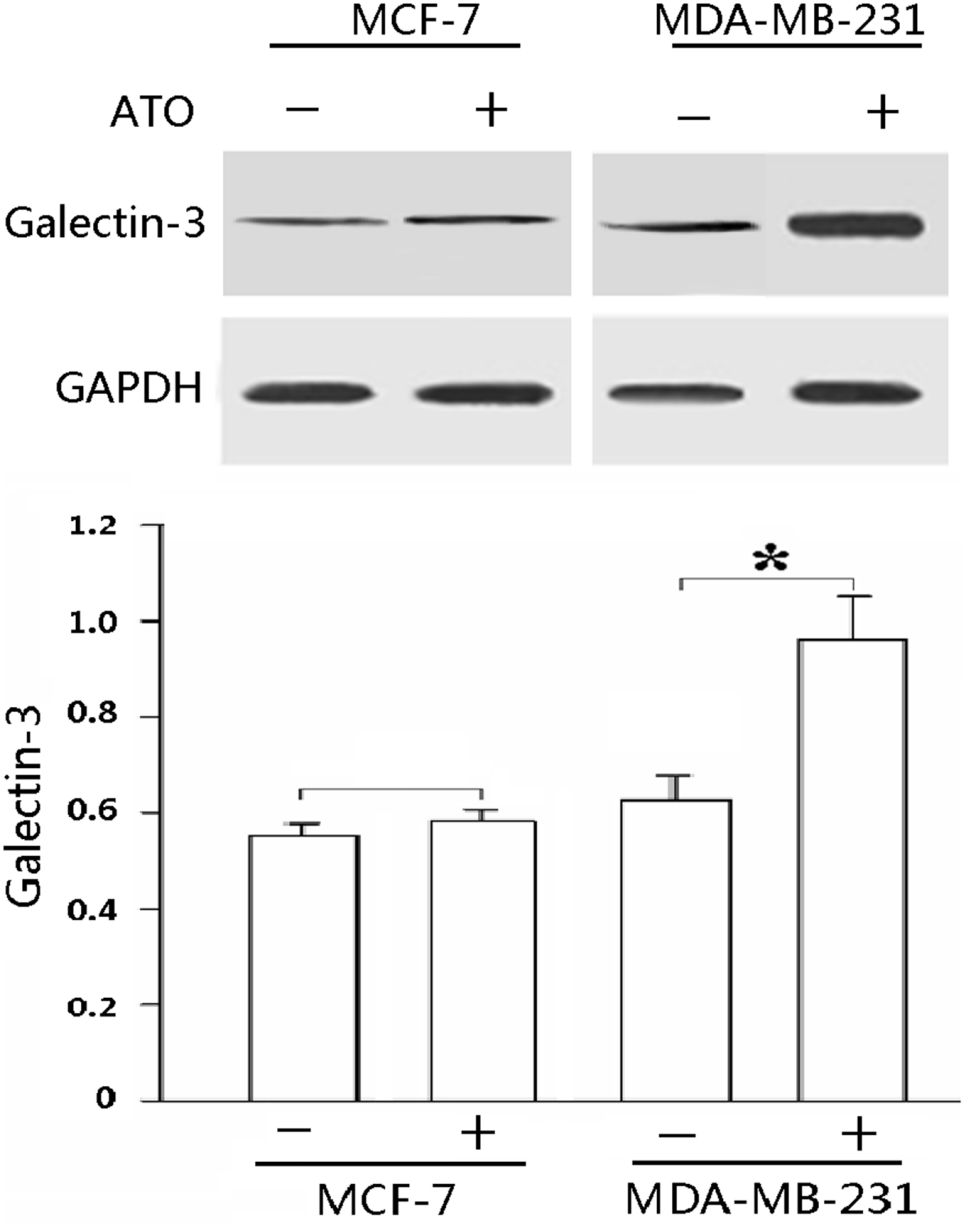
ATO treatment (2.5 µM) significantly increased endogenous galectin-3 expression in MDA-MB-231 cells. Cells were treated with ATO and anti-galectin-3 antibody (1∶1000) was used to detect endogenous galectin-3 proteins. GAPDH was used as loading control. The results shown are the mean of at least 3 independent experiments. *P<0.01.

In order to determine whether galectin-3 expression changed upon ATO-induced apoptosis, we compared the protein level of galectin-3 before and after ATO treatment in MDA-MB-231 and MCF7 cells under hypoxic conditions. Galectin-3 was expressed in both cell types before treatment, but its expression was significantly upregulated after MDA-MB-231 cells were treated with ATO (P<0.01; Figure 3).

### Knockdown of galectin-3 increases the sensitivity of MDA-MB-231 cells to ATO-induced apoptosis

**Figure 4:**
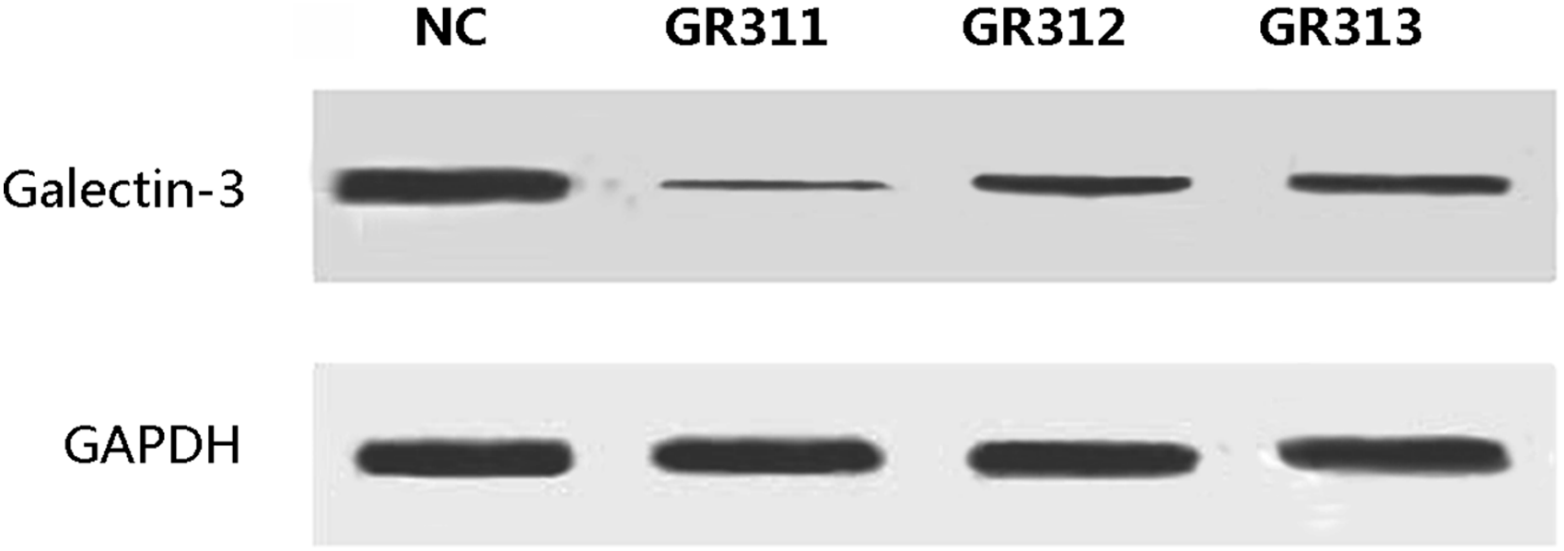
The protein level of galectin-3 was reduced after shRNA treatment. Three independent shRNAs against galectin-3 were used to construct stable cell lines.

**Figure 5:**
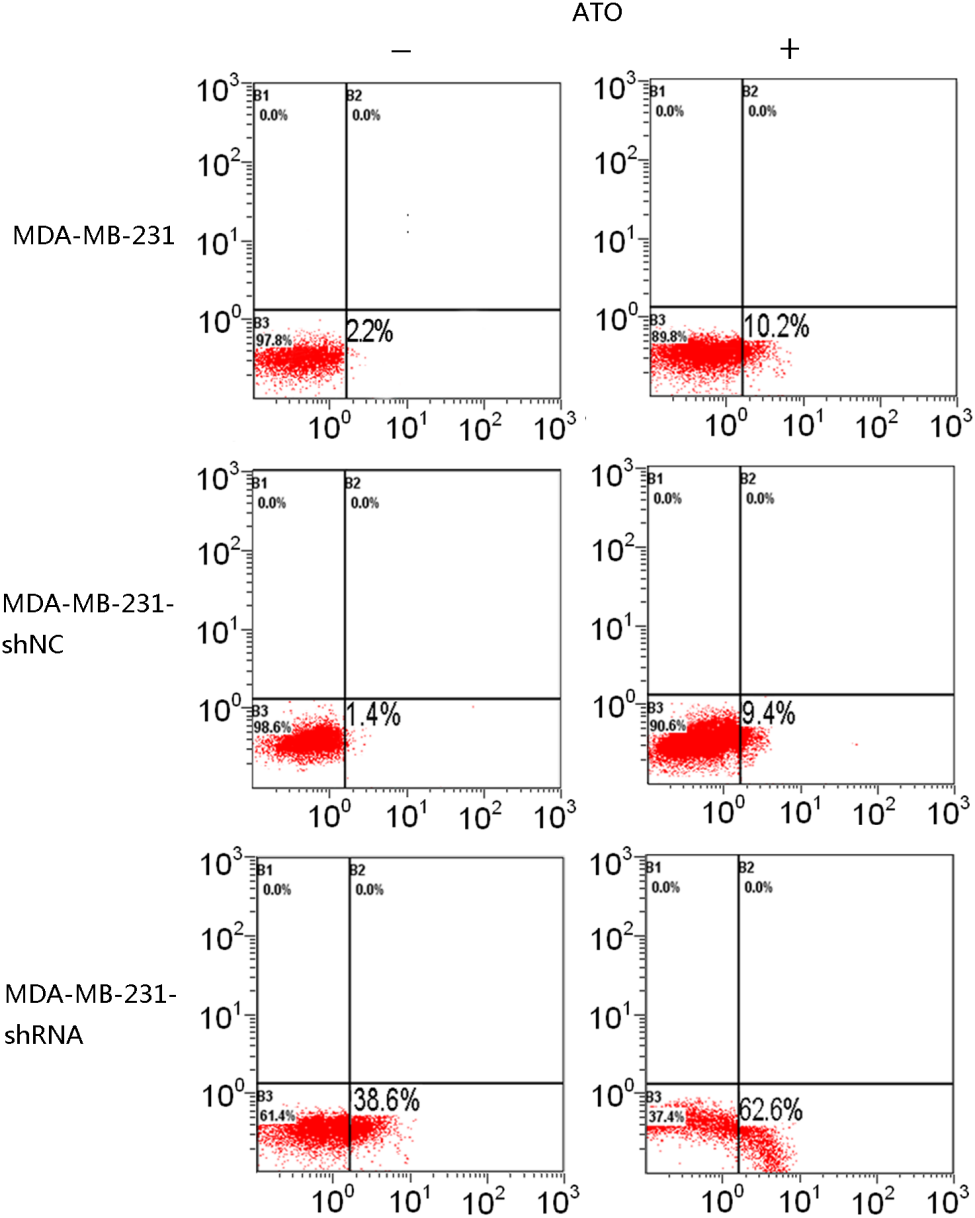
Galectin-3 knockdown sensitized MDA-MB-231 cells to ATO-induced apoptosis. Cells were labeled with annexin V (x-axis) and PI (y-axis), and apoptosis was analyzed using a flow cytometer.

**Figure 6:**
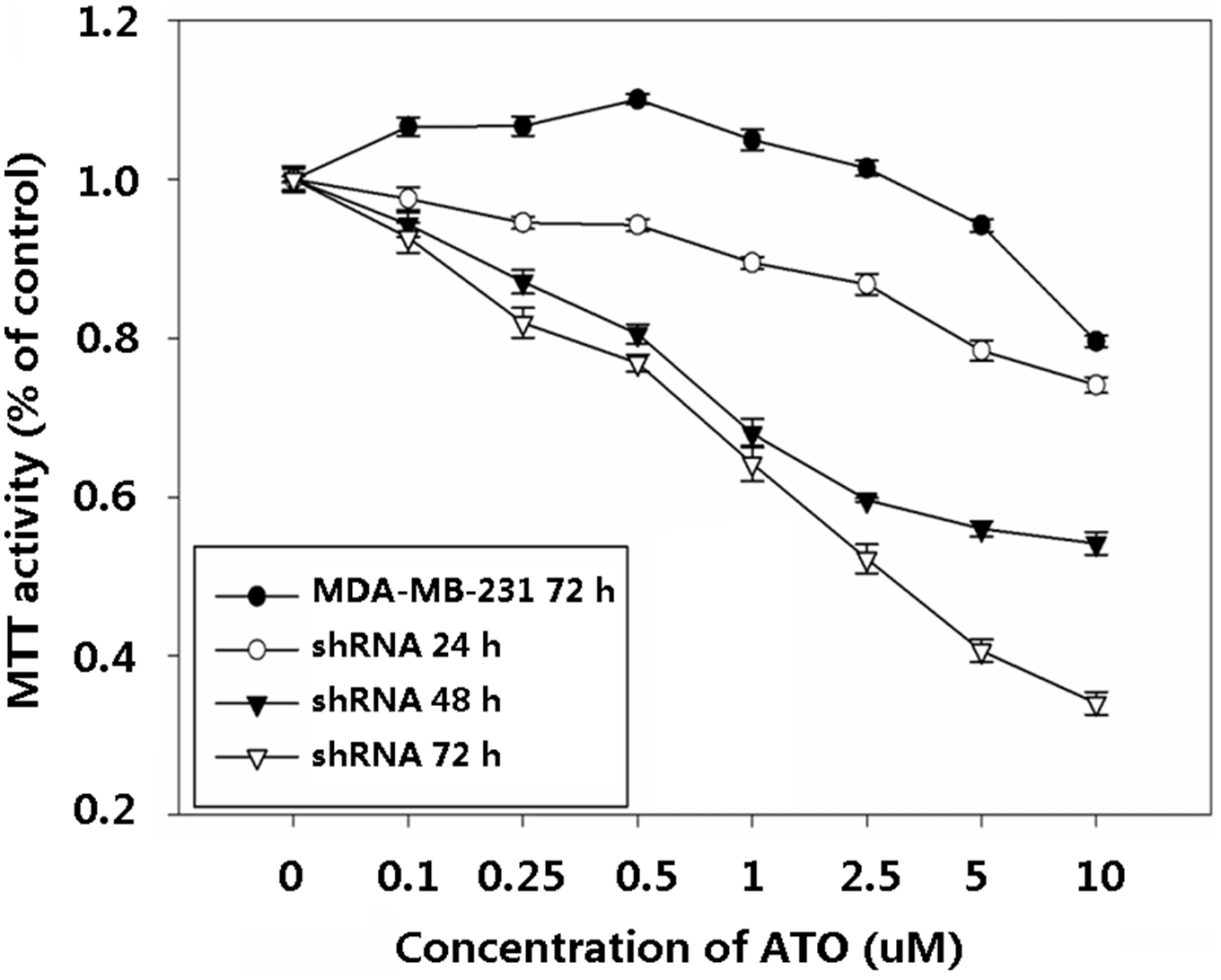
Cell viability was reduced by combined galectin-3 knockdown and ATO treatment.

In order to investigate whether galectin-3 has anti-apoptotic activity in cells treated with ATO, we used shRNA to knockdown its expression in MDA-MB-231 cells. Independent shRNA constructs (GR311, GR312, and GR313) were used to knock down the endogenous galectin-3 transcript, and the galectin-3 protein level was significantly reduced in each case (Figure 4). GR311 was the most effective at reducing galectin-3 expression (to approximately 10% of the control level) and hence was chosen for the following experiments. Control shRNA alone did not induce apoptosis in MDA-MB-231 cells or change the ability of ATO to do so (Figure 5). However, galectin-3-knockdown MDA-MB-231 cells showed dramatically increased apoptosis, about 20-fold more than control group (P<0.05). Interestingly, when galectin-3-knockdown MDA-MB-231 cells were treated with ATO, apoptosis increased further, by about 2-fold (Figure 5), suggesting that galectin-3 inhibition sensitizes MDA-MB-231 cells to ATO-induced apoptosis. To confirm this, galectin-3-knockdown MDA-MB-231 cells were treated with different concentrations of ATO for 24, 48, and 72 h, respectively, and their viability was assessed using the MTT assay. The results showed that cell killing by ATO was considerably enhanced by galectin-3-knockdown. The viability of galectin-3 knockdown MDA-MB-231 cells treated with 0.5 µM ATO for 48 h was equal to that of wild-type MDA-MB-231 cells treated with 10 µM ATO for 72 h (Figure 6). Furthermore, the IC50 of ATO for galectin-3 knockdown MDA-MB-231 cells after 72 h treatment was reduced to 1 µM.

## Discussion

Breast cancer is a clinically heterogeneous disease, for which endocrine therapy and trastuzumab adjuvant treatment have been used successfully to treat patients with ER+ and HER2-overexpressing tumors [13],[14]. Therefore, the expression of these proteins has considerable prognostic significance, and a considerable effort has been made to understand the clinical significance of known markers, to find how they relate to each other, and to discover new ones.

Galectin-3, a member of the β-galactoside-binding lectin family, is thought to have a number of different biological roles, probably including the progression and metastasis of cancer through regulation of homotypic and heterotypic cell-cell and cell-matrix interactions. Furthermore, its expression has been both positively and negatively associated with tumor progression. In vitro studies using MDA-MB-435 and BT-549 breast cancer cell lines have shown a direct association between galectin-3 expression and metastatic and invasive potential [15],[16]. Furthermore, stable transfection of BT-549 (galectin-3 null) or MDA-MB-435 (galectin-3 expressing) cells with sense or anti-sense galectin-3, respectively, caused changes in the tumorigenic phenotype [15],[16].

Based on the results of gene expression profiling analyses, breast cancer has been classified into basal-like, normal breast-like, luminal A, luminal B, and HER2 over-expressing types [17]. Approximately 80–90% of TNC tumors overlap with basal-like breast cancer (BLBC) on the basis of their DNA microarray and immunohistochemical (IHC) profiles and also have a similar clinical behavior to BLBC [18]. There is no effective treatment for TNC as it lacks ER and PR expression, thus precluding hormone treatment, and lacks HER2 expression, precluding trastuzumab treatment [19].

Galectin-3 is preferentially expressed in TNC tumors [7], and likewise, in this study, we found that galectin-3 was predominantly expressed in this subtype. We also showed that ATO, a Food and Drug Administration-approved treatment for refractory acute promyelocytic leukemia (APL) and a proven therapeutic agent for relapsed/refractory multiple myeloma [5],[20]–[22], is a potential therapy for breast cancer. Several mechanisms of action have been proposed for ATO activity, including the induction of apoptosis mediated by reactive oxygen species [22],[23]. Preclinical studies of ATO have demonstrated that it has antitumor activity in murine solid tumor models, including breast, brain, liver, gastric, prostate, renal, and bladder cancer [24],[25]. APL patients are administered 0.16 mg/kg/d of ATO, but this dose is associated with grade 3/4 toxicities such as peripheral neuropathy and hepatic and cardiac toxicity [5],[26]. These dose-limiting toxicities have prevented further dose escalation of ATO in other malignancies.

In this study, we found that ATO could induce apoptosis in the breast cancer derived cell lines MCF-7 and MDA-MB-231 cells. Galectin-3 was expressed in both breast cancer cell lines, but was significantly upregulated in MDA-MB-231 cells after ATO treatment. Galectin-3 is known to have an anti-apoptotic role, and its high expression level in breast cancer cells might contribute to the inhibition of ATO-induced apoptosis. As the MCF-7 cell lines are ER^+^PR^+^ and the MDA-MB-231 cell lines are triple negative, the ATO may be more relevant in TNC since galectin-3 was found to be higher in the TNC cases.

In summary, our findings show that galectin-3 is consistently expressed in TNC and is associated with specific clinicopathological and immunohistochemical characteristics. Galectin-3 might be a subtype-specific marker for breast cancer, and a potential target in overcoming resistance to chemotherapy.
