# Correction: Galectin-3 as a Marker and Potential Therapeutic Target in Breast Cancer

**DOI:** 10.1371/journal.pone.0116994

**Published:** 2015-01-26

**Authors:** The PLOS ONE Staff

Minna Luo, Xinhan Zhao, Xi Liang, Dan Wang, Guanglei Chen, Xin Gu, Chao Duan, and Huizi Gu should be included in the author byline of this article. The publisher apologizes for the errors. The correct author byline and affiliations are as follows:

Hao Zhang^1¶^, Minna Luo^2¶^, Xi Liang^1¶^, Dan Wang^1¶^, Xin Gu^3¶^, Chao Duan^4¶^, Huizi Gu^5^, Guanglei Chen^1^, Xinhan Zhao^2^, Zuowei Zhao^1^*, Caigang Liu^1^*

^1^ Breast Disease and Reconstruction Center, Breast Cancer Key Lab of Dalian, the Second Hospital of Dalian Medical University, Dalian, China

^2^ Department of Oncology, The First Affiliated Hospital, College of Medicine, Xi’an Jiaotong University, Xi’an, China.

^3^ Department of Head and Neck Surgery, the Affiliated Tumor Hospital of Harbin Medical University, Harbin, China

^4^ Department of Cardiothoracic, Benxi Central Hospital, Benxi, China 117000

^5^ Department of Internal Neurology, the Second Hospital of Dalian Medical University, Dalian, China 116023

* Email: angel-s205@163.com

¶ These authors are co-first authors on this work.

The correct citation is: Zhang H, Luo M, Liang X, Wang D, Gu X (2014) Galectin-3 as a Marker and Potential Therapeutic Target in Breast Cancer. PLoS ONE 9(9): e103482. doi: 10.1371/journal.pone.0103482

The correct author contributions are: Conceived and designed the experiments: HZ CL ZZ. Performed the experiments: HZ XL. Analyzed the data: HZ HG. Contributed reagents/materials/analysis tools: ML XZ XG DW CD GC XL. Wrote the paper: HZ CL CD ZZ. Drafted the manuscript: HZ CL ZZ. Helped to draft the manuscript: CD. Read and approved the final manuscript: HZ ML XL DW XG CD HG GC XZ ZZ CL.
