# Correction: Galectin-3 as a Marker and Potential Therapeutic Target in Breast Cancer

**DOI:** 10.1371/journal.pone.0232166

**Published:** 2020-04-16

**Authors:** Hao Zhang, Minna Luo, Xi Liang, Dan Wang, Xin Gu, Chao Duan, Huizi Gu, Guanglei Chen, Xinhan Zhao, Zuowei Zhao, Caigang Liu

After publication of this article [1], the authors notified *PLOS ONE* of concerns about the results published in Figs 2 and 5. They explained that experiments for Figs 2 and 5 in [1] had been conducted by an external third-party company, and that initial replication efforts in the authors’ laboratory had not reproduced the published findings.

Subsequently, the authors replicated these experiments again and obtained results that support the published findings. In this Correction, the authors provide the replication results along with the available data from these experiments in S1–S6 Files. The raw flow cytometry (.fcs) data files from the replication experiments are no longer available. Overall, the replication results show moderate differences from the original published figures [1] in the percentages of apoptotic cells. The differences may stem from usage of different passages of cells; the authors previously expressed a concern about MDA-MB-231 cells that grew slowly, so for the replication experiments they used cells from freshly thawed vials of MDA-MB-231 and MCF-7 cells. Although these cells displayed similar levels of Galectin-3 expression they had varying frequencies of spontaneous apoptotic cells, but slightly lower sensitivity to ATO-induced apoptosis, compared to that of previous MDA-MB-231 cells and MCF-7 cells used for experiments in the article (compare S1 File versus the published version of Fig 2 in [1]). Although the replication data do not perfectly match the published data, they indicate that:

- treatment with ATO up-regulated Galectin-3 expression in MDA-MB-231 but not in MCF-7 cells, consistent with Fig 3 in [1];
- treatment with ATO increased the frequency of apoptotic MCF-7 and MDA-MB-231 cells, consistent with the data in Fig 2 of [1]; and
- Galectin-3 silencing increased the frequency of apoptotic MDA-MB-231 cells and sensitized them to ATO-induced apoptosis, consistent with the data in Fig 5 in [1].

Therefore, the replication data, together with human histological data, support the conclusion that Galectin-3 may be a biomarker and therapeutic target of triple-negative breast cancer.

In Fig 3 of the original article [1], there is a vertical line suggestive of image splicing between the two lanes of the MDA-MB-231 Galectin-3 blot. The original data supporting the published figure are no longer available and so the authors were unable to clarify the reason for this image issue. The authors provide here results from replication experiments that were done in triplicate, along with the underlying image and quantification data (S7 File).

The authors also provide here (S8 File) replication data S4 Fig, as the original data underlying this figure are no longer available.

In the replication experiments for the updated versions of Figs 2, 3, 4 and 5, the authors repeated the experiments using the same methods described in the published article.

In addition, it was noted that in Table 1, *P* was reported as 0.000 in three instances. A corrected version of Table 1 is included with this Correction in which these values are reported as <0.001. Data underlying the results in Table 1 are in S9 File.

**Table 1:** Galectin-3 expression and clinicopathological features (n = 1187). Clinicopathological data and Galectin-3 expression levels are the same as that in Table 1 of the published article [1]. All the *p*-values of 0 in the Table 1 of the published article are revised to <0.001. DCIS, ductal carcinoma in situ; IDC, invasive ductal carcinoma. χ^2^-test was used to assess the difference in the percentages of cases with or without Galectin-3 expression in a given clinicopathological parameter. (n = 1187) See S12 File for details of the statistical analyses.

Variables	Galectin-3^–^ (%)	Galectin-3^+^ (%)	*P* value
**Age**			0.023
<35Y	139 (17.4)	89 (22.9)	
≥35 Y	660 (82.6)	299 (77.1)	
**Tumor size**			< 0.001
T1	156 (19.5)	47 (12.1)	
T2	557 (69.7)	318 (82)	
T3	76 (9.5)	17 (4.4)	
T4	10 (1.3)	6 (1.5)	
**Histological grade**			< 0.001
I	72 (9)	43 (11.1)	
II	694 (86.9)	78 (20.1)	
III	33 (4.1)	267 (68.8)	
**Tumor stage**			0.023
DCIS	154 (19.3)	97 (25)	
IDC	645 (80.7)	291 (75)	
**Metastatic nodes**			0.001
positive	356 (44.6)	213 (54.9)	
negative	443 (55.4)	175 (45.1)	
**Her-2 status**			0.251
positive	225 (28.2)	97 (25)	
negative	574 (71.8)	291 (75)	
**Triple-negative breast cancer**			< 0.001
yes	95 (11.9)	131 (33.8)	
no	704 (88.1)	257 (66.2)	

DCIS = ductal carcinoma in situ, IDC = invasive ductal carcinoma.

χ^2^-test was used to assess the difference of percentages of cases with or without Galectin-3 expression in clinicopathological parameter.

*P* < 0.05 was considered statistically significant.

The authors also provide with this Correction a new version of Table 2. Note that the Materials and Methods section in [1] describing statistical analysis methods used for this table is incorrect. Rather than a logistic regression, a Cox proportional hazards (PH) model was used to determine which factors were predictive of time to post-operative distant metastasis. The corrected version of Table 2 reports results of a reanalysis in which prognostic factors were entered into a multivariate Cox regression model including age, tumor size, histological grade, tumor stage, lymph node metastasis, Galectin-3, and triple-negative breast cancer. In addition, the reanalysis used data from a longer follow-up period (to December 2017, versus December 2012 in [1]). Several factors, including age, tumor stage, and Galectin-3, show significant associations with post-operative distant metastasis in the new analyses (Table 2) but did not emerge as statistically significant in the original analyses [1]. However, for triple-negative breast cancers, the authors observed a larger *p* value in the reanalysis than in the originally reported analyses. The strong significance of Galectin-3 in the reanalysis (*p*<0.001) further supports the conclusion that Galectin-3 expression is an independent prognostic factor of time to post-operative distant metastasis. In the Galectin-3 expression and survival subsection of the Results in [1], the results statement regarding the outcome of analyses in Table 2 states, “A subsequent multivariate analysis revealed that histological grade, lymph node metastasis, and tumor size were significantly associated with post-operative distant metastasis . . .” In light of the reanalysis reported in this notice, this statement should be updated to: “Using Cox regression, age, tumor size, histology, tumor stage, lymph node metastasis and Galectin-3 expression were significantly associated with time to post-operative distant metastasis in a multivariate model also containing TNBC.” Data underlying the results in the updated version of Table 2 are in S10 File.

**Table 2:** Multivariate analysis of the factors related to post-operative distant metastasis. Multivariate analysis of the factors related to post-operative distant metastasis. The results shown in Table 2 of this Correction reflect reanalysis of data for the same participants as in [1] but over a longer follow-up period (up to December 2017 versus up to December 2012). CI, confidence interval. See S13 File for details of the statistical analyses.

Characteristic	Hazard Ratios	95% CI for Hazard Ratios	P value
Age	0.086	0.034–0.217	< 0.001
Tumor size	2.521	1.981–3.209	< 0.001
Histological grade	1.918	1.346–2.733	< 0.001
Tumor stage	3.277	1.435–7.487	0.0050
Lymph node metastasis	4.607	3.572–5.943	< 0.001
Galectin-3	2.211	1.607–3.042	< 0.001
Triple-negative breast cancer	0.855	0.619–1.181	0.3420

CI = confidence interval.

The underlying data S6 Fig in [1] are provided here in S11 File.

A member of *PLOS ONE*’s Editorial Board and an external reviewer reviewed the updated figures and confirmed that they support the results and conclusions reported in the article.

## Supporting information

S1 File Replication results for Fig 2, showing ATO treatment induced apoptosis of breast cancer cells. MDA-MB-231 and MCF-7 cells were treated with, or without, ATO (2.5 μM) for 48 h. The cells were stained with PI and annexin V, followed by flow cytometry analysis. Data are representative charts or expressed as the mean ± SD of each group from recently repeated three experiments. There was no significant difference between the new data and the data in the Fig 2 of the published article.
(TIF)

S2 File Raw data supporting the results in S1 File (replication data S2 Fig). (PDF)

S3 File Raw data supporting the statistical results reported in S1 File. (XLS)

S4 File Replication results for Fig 5, showing Galectin-3 knockdown sensitized MDA-MB-231 cells to ATO-induced apoptosis. MDA-MB-231 cells were transfected with, or without, control siRNA or Galectin-3 specific siRNA for 24 h and treated with ATO (2.5 μM) for 48 h. Subsequently, the cells were stained with FITC-Annexin V and PI. The percentages of apoptotic cells in the different groups of cells were determined by flow cytometry. Data are representative charts or expressed as the mean ± SD of each group of cells from three recently repeated experiments. There was no significant difference between the new data and the data in the Fig 5 of the published article.
(TIF)

S5 File Raw data supporting the results in S4 File (replication data S5 Fig). (PDF)

S6 File Raw data supporting the statistical results reported in S4 File. (XLS)

S7 File Replication results for Fig 3, showing ATO treatment significantly increased endogenous Galectin-3 expression in MDA-MB-231 cells. MDA-MB-231 cells were treated with, or without, ATO (2.5 μM) for 48 h and the relative levels of Galectin-3 to GAPDH protein expression were determined by western blot using anti-Galectin-3 antibody. Data in Fig 3.tif (expressed as the mean ± SD of each group of cells) and the Excel file were obtained by densitometric analysis of western blot results from three experiments for which image data are provided. There was no significant difference between the new data and the data in the Fig 3 of the published article. Image file name suffixes (“-1”, “-2”, “-3”) indicate the replicate number, i.e. “Fig 3” and “Fig 3-GAPDH” files with corresponding suffixes present data from the same experiment.
(ZIP)

S8 File Replication data S4 Fig, including raw images and quantitative densitometry and statistical analysis data from western blot experiments examining Galectin-3 expression in MDA-MB-231 cells after Galectin-3 silencing. “Fig 4” and “Fig 4-GAPDH” files with corresponding suffixes present data from the same experiment.
(ZIP)

S9 File Raw data file S1 Table. (XLSX)

S10 File Raw data file supporting the updated version of Table 2. (XLSX)

S11 File Underlying data for Fig 6 in [1]. (XLS)

S12 File Statistics of Table 1. (DOCX)

S13 File Statistics of Table 2. (DOCX)

## References

[1] Zhang H, Liang X, Duan C, Liu C, Zhao Z (2014) Galectin-3 as a Marker and Potential Therapeutic Target in Breast Cancer. PLoS ONE 9(9): e103482 10.1371/journal.pone.0103482 25254965 PMC4177814

