# Supplementary figures and images for: Correction: Galectin-3 as a Marker and Potential Therapeutic Target in Breast Cancer
Source: PLoS One. 2020 Apr 16;15(4):e0232166. doi: 10.1371/journal.pone.0232166 (PMC7162513; doi:10.1371/journal.pone.0232166)

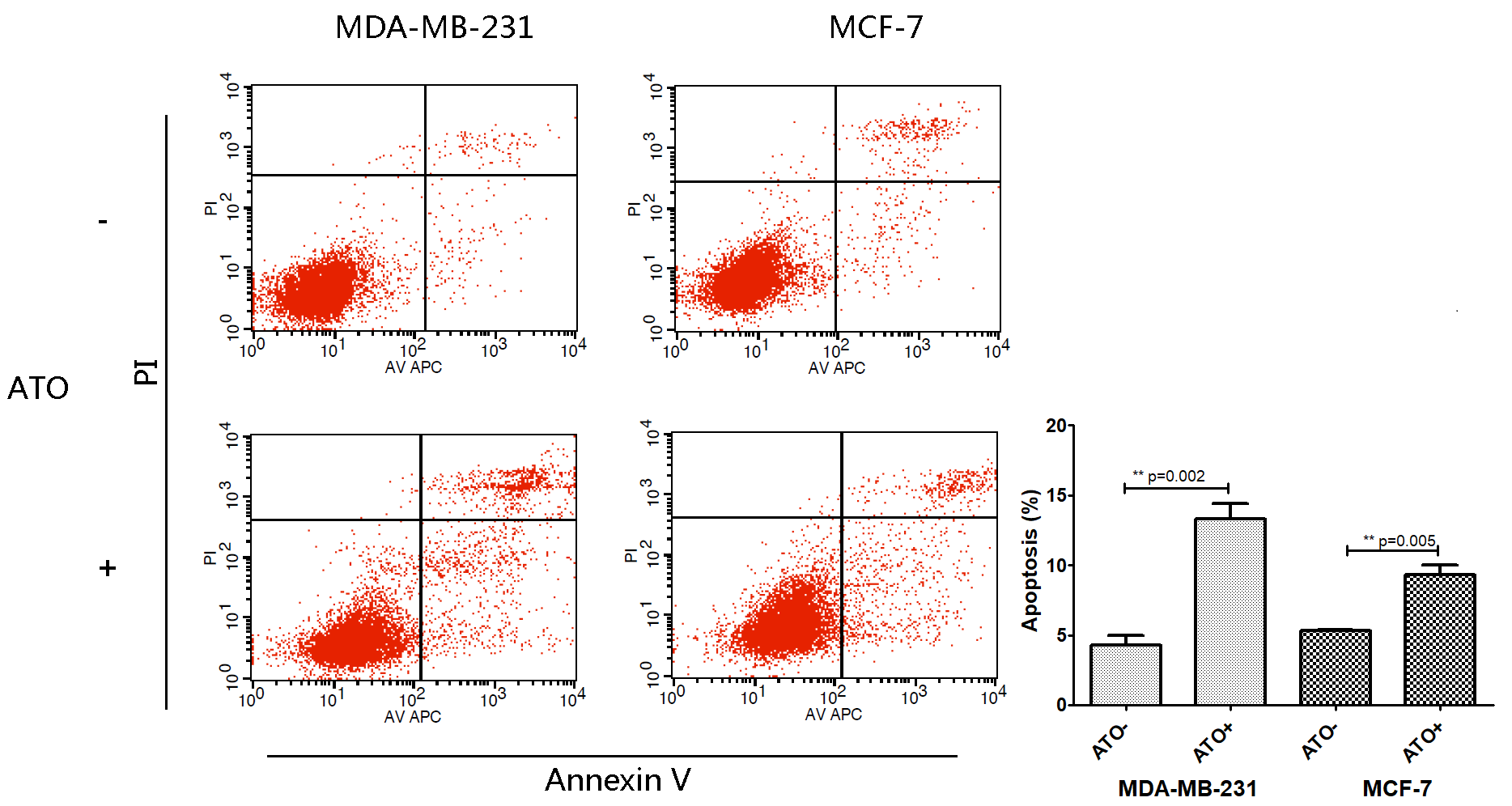

Supplement: S1 File — MDA-MB-231 and MCF-7 cells were treated with, or without, ATO (2.5 μM) for 48 h. The cells were stained with PI and annexin V, followed by flow cytometry analysis. Data are representative charts or expressed as the mean ± SD of each group from recently repeated three experiments. There was no significant difference between the new data and the data in the Fig 2 of the published article. (TIF) [file pone.0232166.s001.tif]

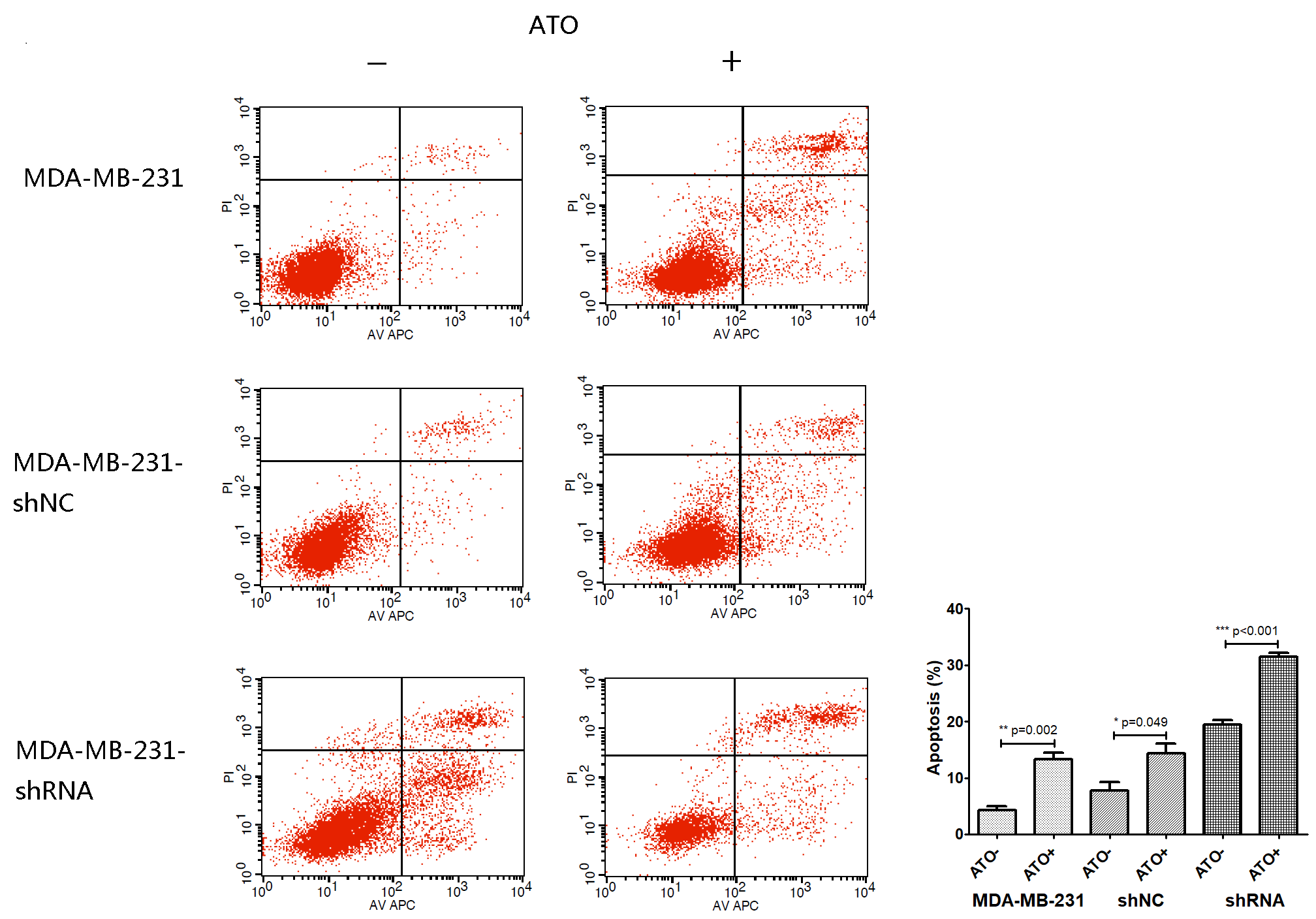

Supplement: S4 File — MDA-MB-231 cells were transfected with, or without, control siRNA or Galectin-3 specific siRNA for 24 h and treated with ATO (2.5 μM) for 48 h. Subsequently, the cells were stained with FITC-Annexin V and PI. The percentages of apoptotic cells in the different groups of cells were determined by flow cytometry. Data are representative charts or expressed as the mean ± SD of each group of cells from three recently repeated experiments. There was no significant difference between the new data and the data in the Fig 5 of the published article. (TIF) [file pone.0232166.s004.tif]

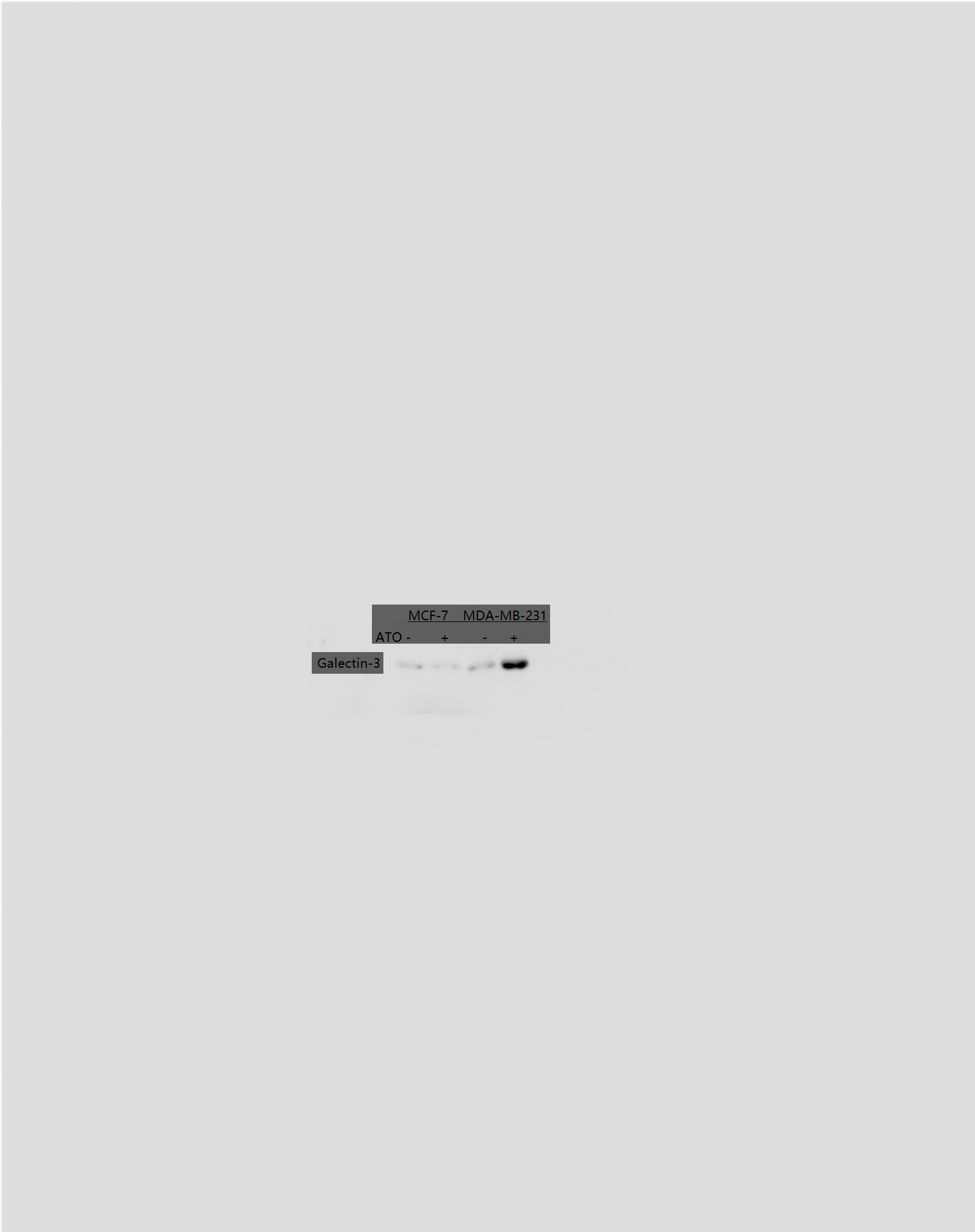

Supplement: S7 File — MDA-MB-231 cells were treated with, or without, ATO (2.5 μM) for 48 h and the relative levels of Galectin-3 to GAPDH protein expression were determined by western blot using anti-Galectin-3 antibody. Data in Fig 3.tif (expressed as the mean ± SD of each group of cells) and the Excel file were obtained by densitometric analysis of western blot results from three experiments for which image data are provided. There was no significant difference between the new data and the data in the Fig 3 of the published article. Image file name suffixes (“-1”, “-2”, “-3”) indicate the replicate number, i.e. “Fig 3” and “Fig 3-GAPDH” files with corresponding suffixes present data from the same experiment. (ZIP) [file pone.0232166.s007.zip › Figure 3-1.tif]

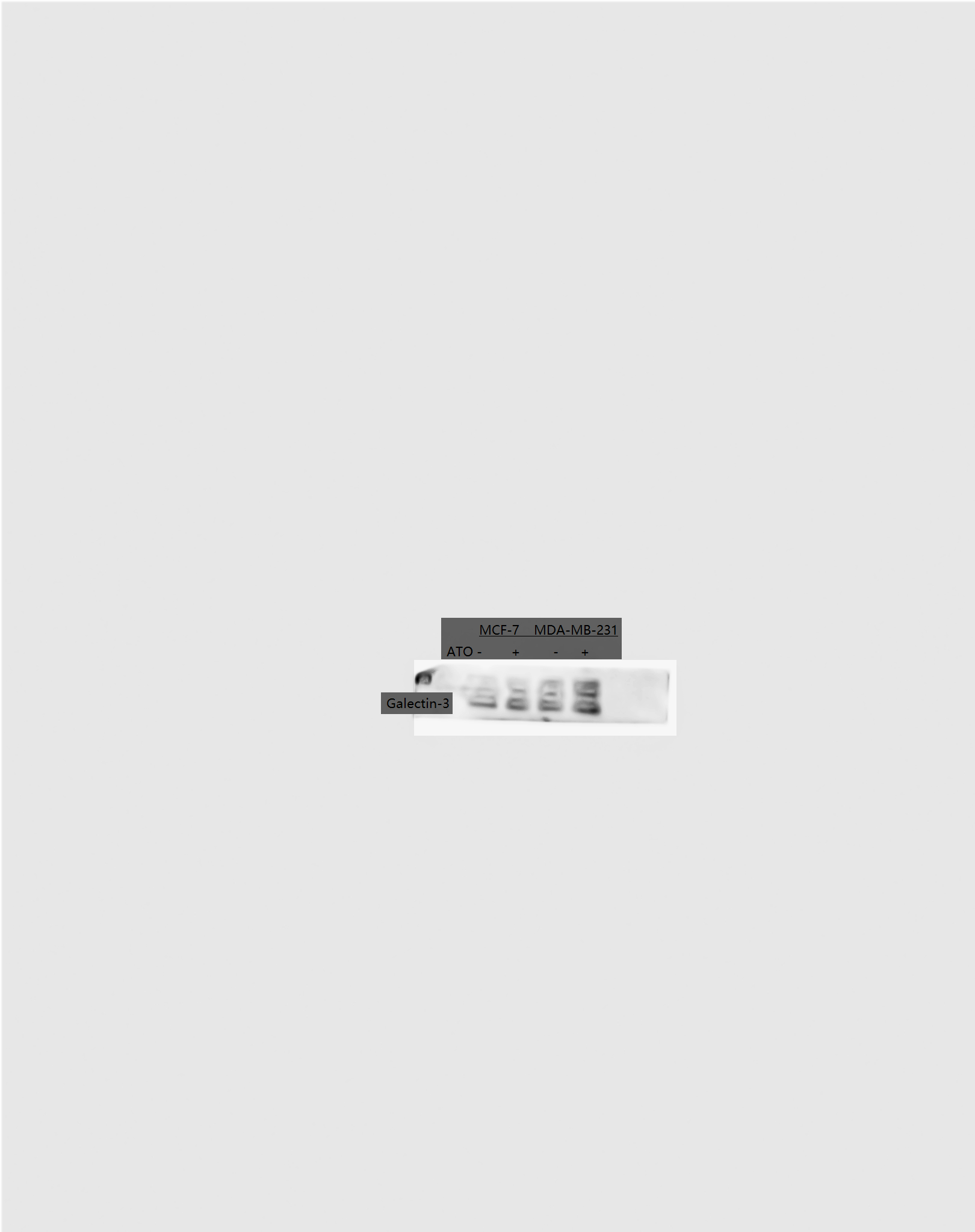

Supplement: S7 File — MDA-MB-231 cells were treated with, or without, ATO (2.5 μM) for 48 h and the relative levels of Galectin-3 to GAPDH protein expression were determined by western blot using anti-Galectin-3 antibody. Data in Fig 3.tif (expressed as the mean ± SD of each group of cells) and the Excel file were obtained by densitometric analysis of western blot results from three experiments for which image data are provided. There was no significant difference between the new data and the data in the Fig 3 of the published article. Image file name suffixes (“-1”, “-2”, “-3”) indicate the replicate number, i.e. “Fig 3” and “Fig 3-GAPDH” files with corresponding suffixes present data from the same experiment. (ZIP) [file pone.0232166.s007.zip › Figure 3-2.tif]

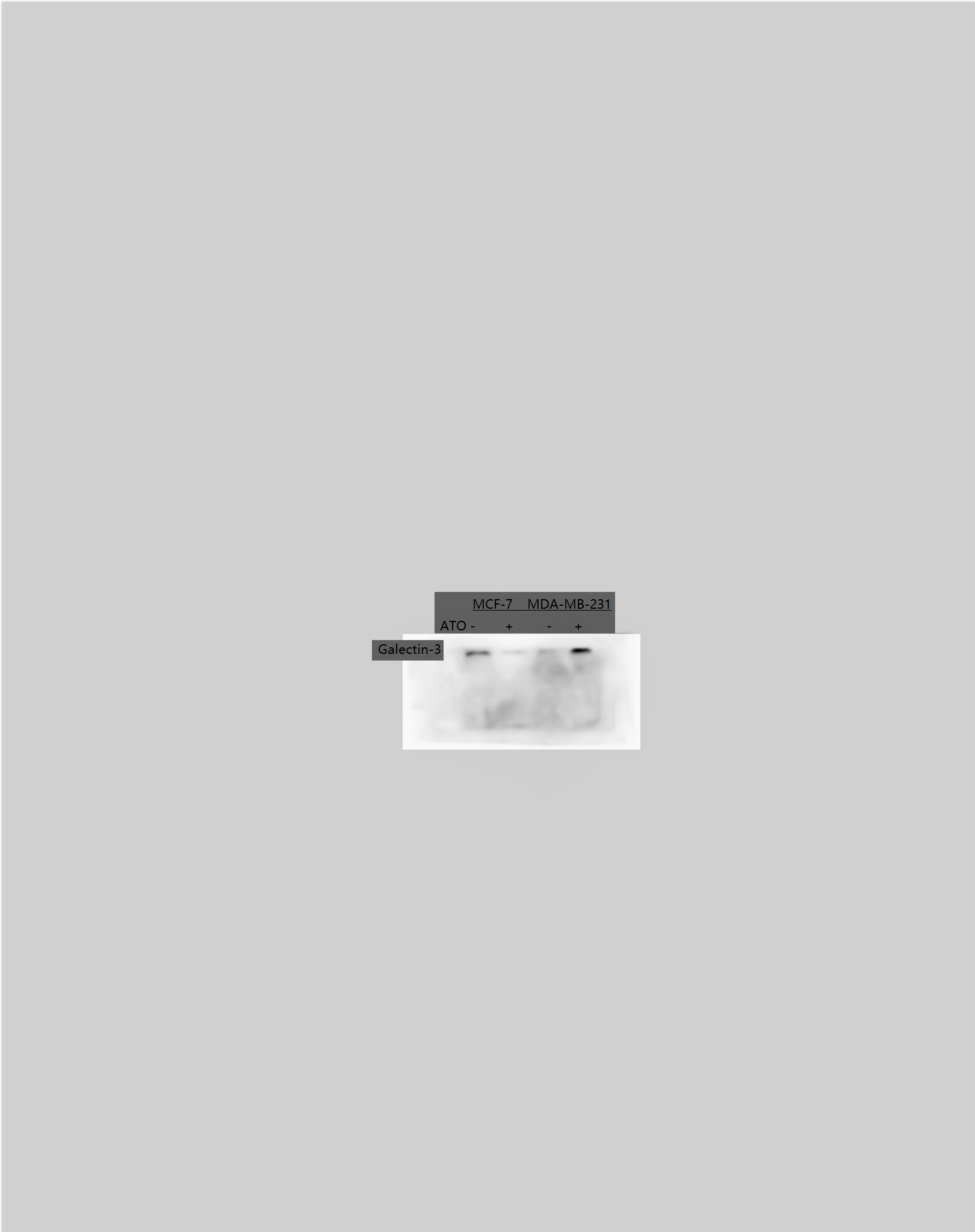

Supplement: S7 File — MDA-MB-231 cells were treated with, or without, ATO (2.5 μM) for 48 h and the relative levels of Galectin-3 to GAPDH protein expression were determined by western blot using anti-Galectin-3 antibody. Data in Fig 3.tif (expressed as the mean ± SD of each group of cells) and the Excel file were obtained by densitometric analysis of western blot results from three experiments for which image data are provided. There was no significant difference between the new data and the data in the Fig 3 of the published article. Image file name suffixes (“-1”, “-2”, “-3”) indicate the replicate number, i.e. “Fig 3” and “Fig 3-GAPDH” files with corresponding suffixes present data from the same experiment. (ZIP) [file pone.0232166.s007.zip › Figure 3-3.tif]

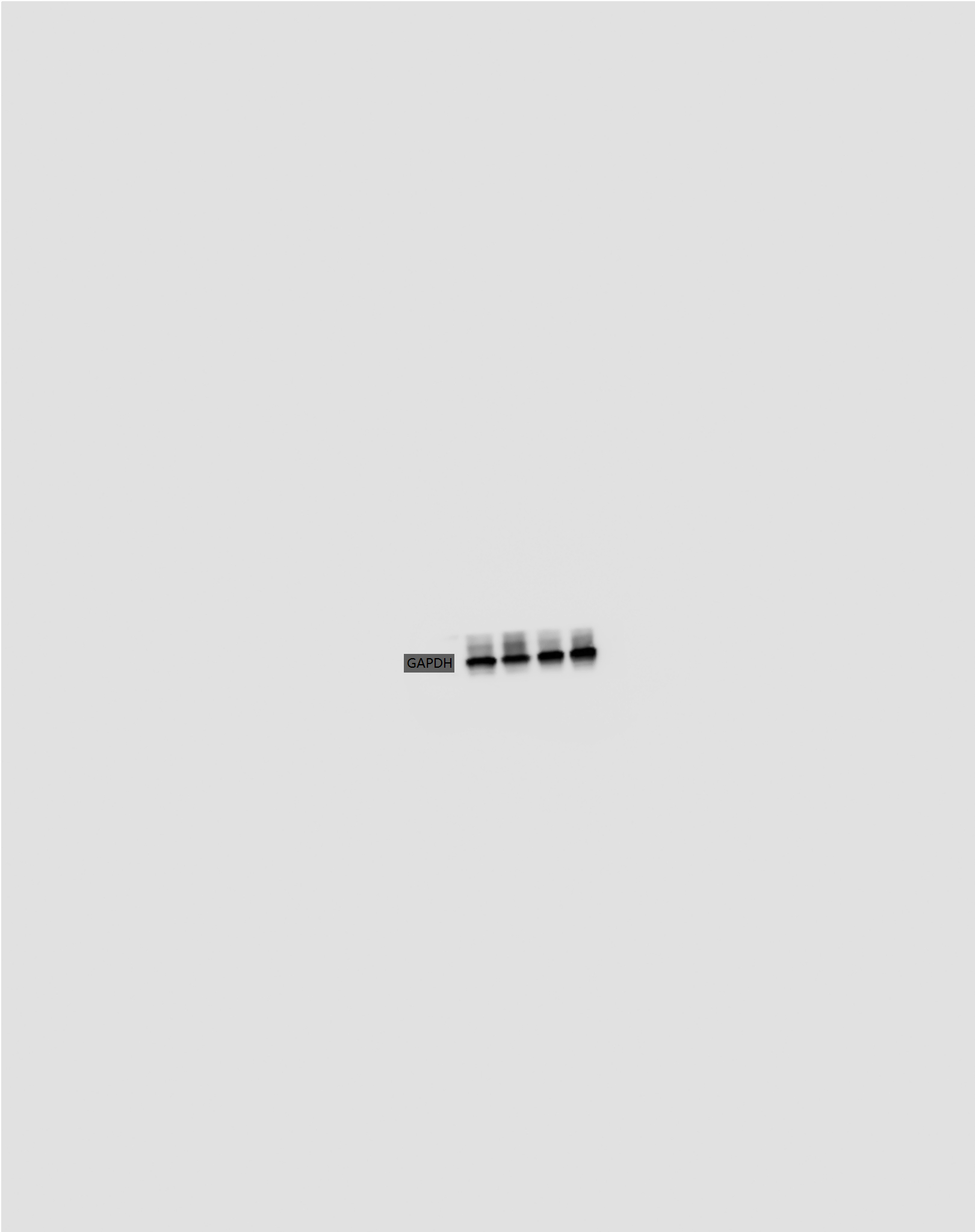

Supplement: S7 File — MDA-MB-231 cells were treated with, or without, ATO (2.5 μM) for 48 h and the relative levels of Galectin-3 to GAPDH protein expression were determined by western blot using anti-Galectin-3 antibody. Data in Fig 3.tif (expressed as the mean ± SD of each group of cells) and the Excel file were obtained by densitometric analysis of western blot results from three experiments for which image data are provided. There was no significant difference between the new data and the data in the Fig 3 of the published article. Image file name suffixes (“-1”, “-2”, “-3”) indicate the replicate number, i.e. “Fig 3” and “Fig 3-GAPDH” files with corresponding suffixes present data from the same experiment. (ZIP) [file pone.0232166.s007.zip › Figure 3-GAPDH-1.tif]

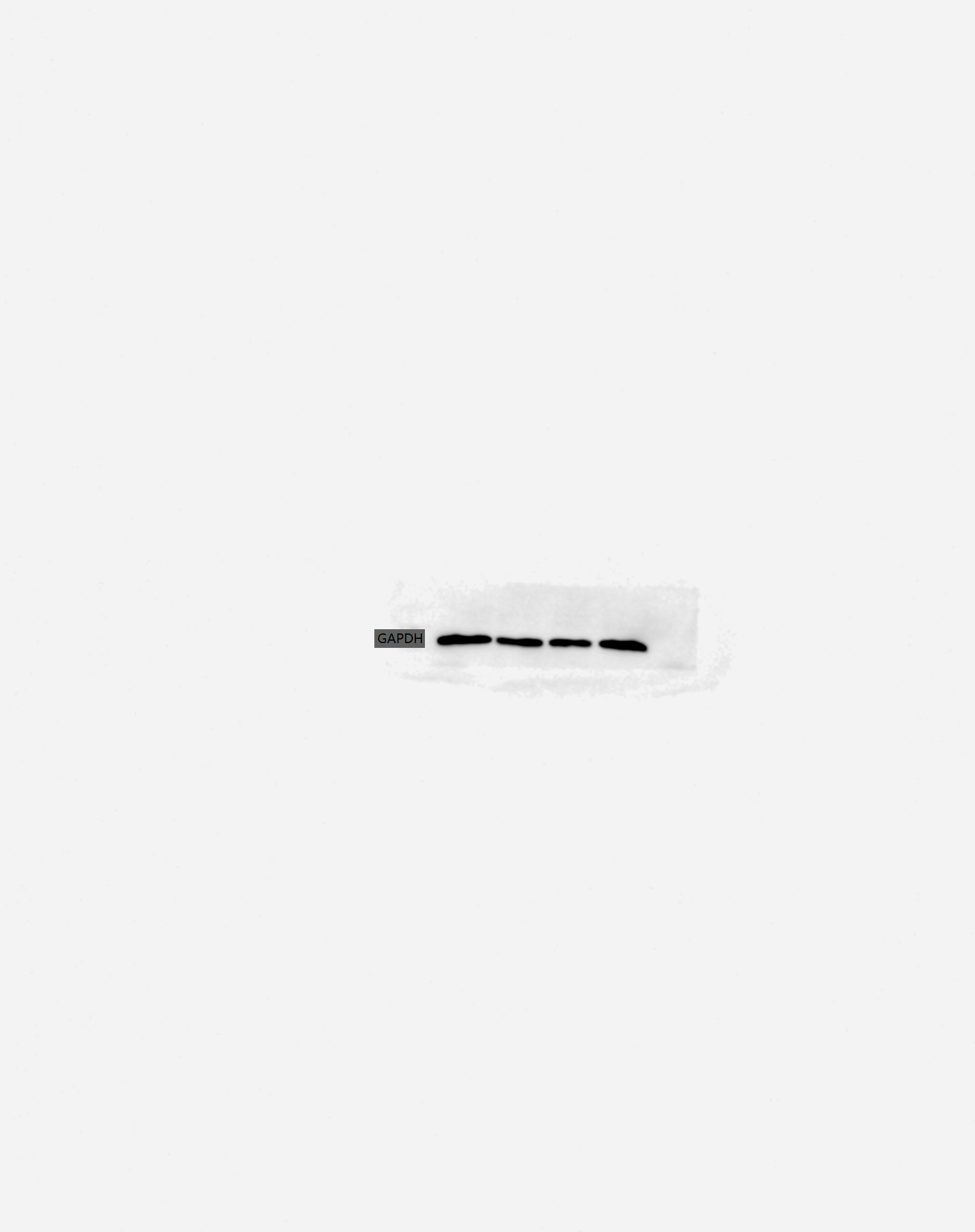

Supplement: S7 File — MDA-MB-231 cells were treated with, or without, ATO (2.5 μM) for 48 h and the relative levels of Galectin-3 to GAPDH protein expression were determined by western blot using anti-Galectin-3 antibody. Data in Fig 3.tif (expressed as the mean ± SD of each group of cells) and the Excel file were obtained by densitometric analysis of western blot results from three experiments for which image data are provided. There was no significant difference between the new data and the data in the Fig 3 of the published article. Image file name suffixes (“-1”, “-2”, “-3”) indicate the replicate number, i.e. “Fig 3” and “Fig 3-GAPDH” files with corresponding suffixes present data from the same experiment. (ZIP) [file pone.0232166.s007.zip › Figure 3-GAPDH-2.tif]

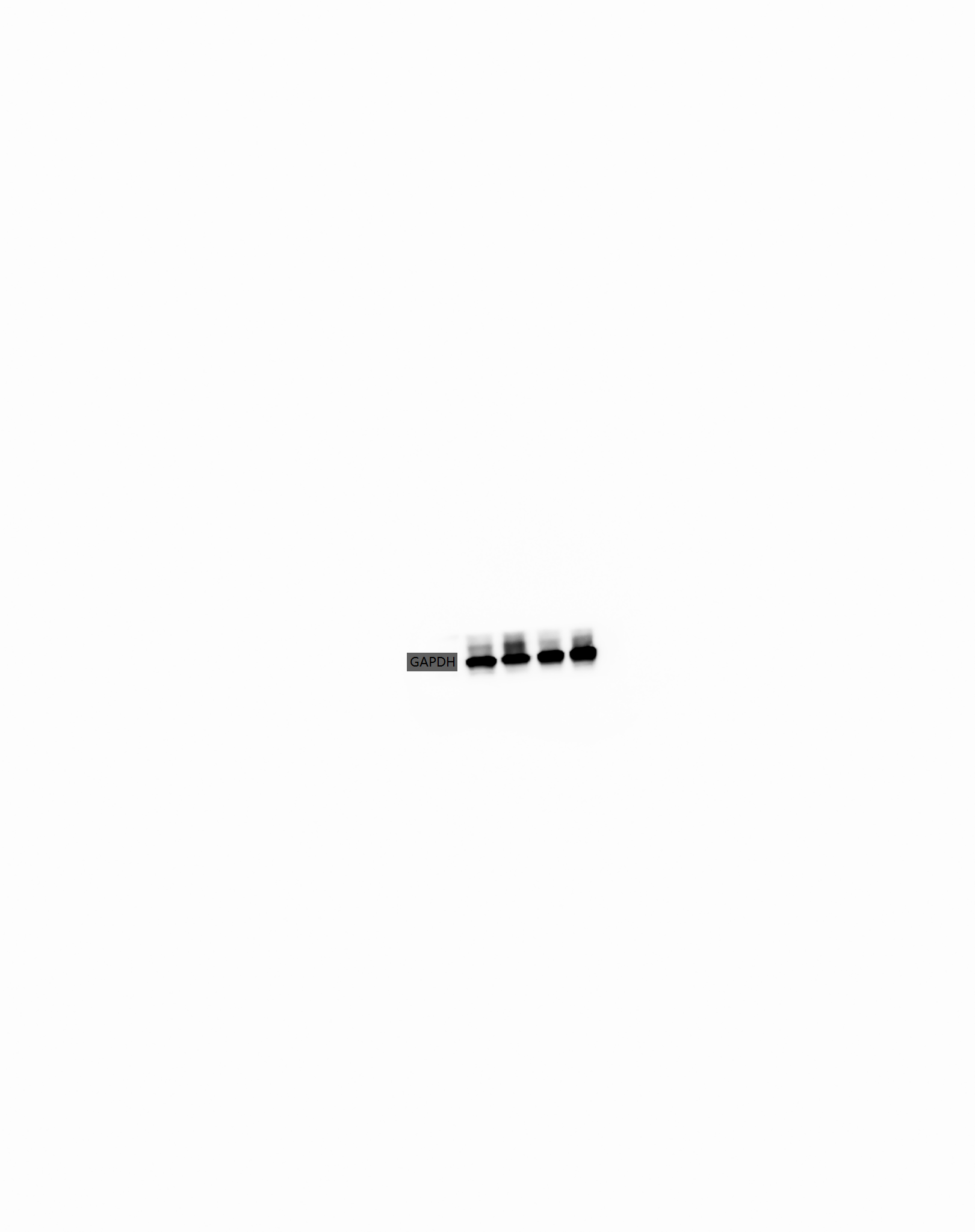

Supplement: S7 File — MDA-MB-231 cells were treated with, or without, ATO (2.5 μM) for 48 h and the relative levels of Galectin-3 to GAPDH protein expression were determined by western blot using anti-Galectin-3 antibody. Data in Fig 3.tif (expressed as the mean ± SD of each group of cells) and the Excel file were obtained by densitometric analysis of western blot results from three experiments for which image data are provided. There was no significant difference between the new data and the data in the Fig 3 of the published article. Image file name suffixes (“-1”, “-2”, “-3”) indicate the replicate number, i.e. “Fig 3” and “Fig 3-GAPDH” files with corresponding suffixes present data from the same experiment. (ZIP) [file pone.0232166.s007.zip › Figure 3-GAPDH-3.tif]

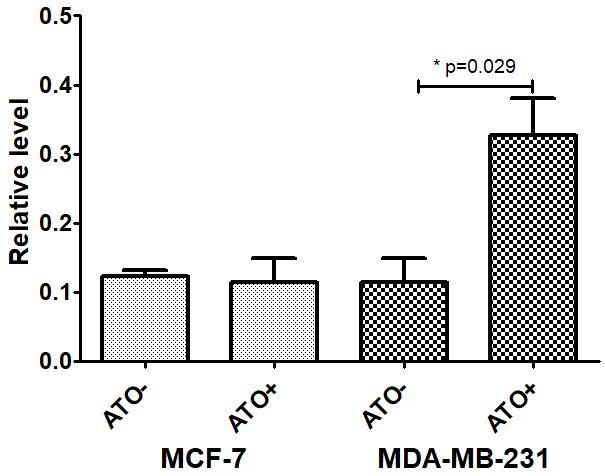

Supplement: S7 File — MDA-MB-231 cells were treated with, or without, ATO (2.5 μM) for 48 h and the relative levels of Galectin-3 to GAPDH protein expression were determined by western blot using anti-Galectin-3 antibody. Data in Fig 3.tif (expressed as the mean ± SD of each group of cells) and the Excel file were obtained by densitometric analysis of western blot results from three experiments for which image data are provided. There was no significant difference between the new data and the data in the Fig 3 of the published article. Image file name suffixes (“-1”, “-2”, “-3”) indicate the replicate number, i.e. “Fig 3” and “Fig 3-GAPDH” files with corresponding suffixes present data from the same experiment. (ZIP) [file pone.0232166.s007.zip › Figure 3.tif]

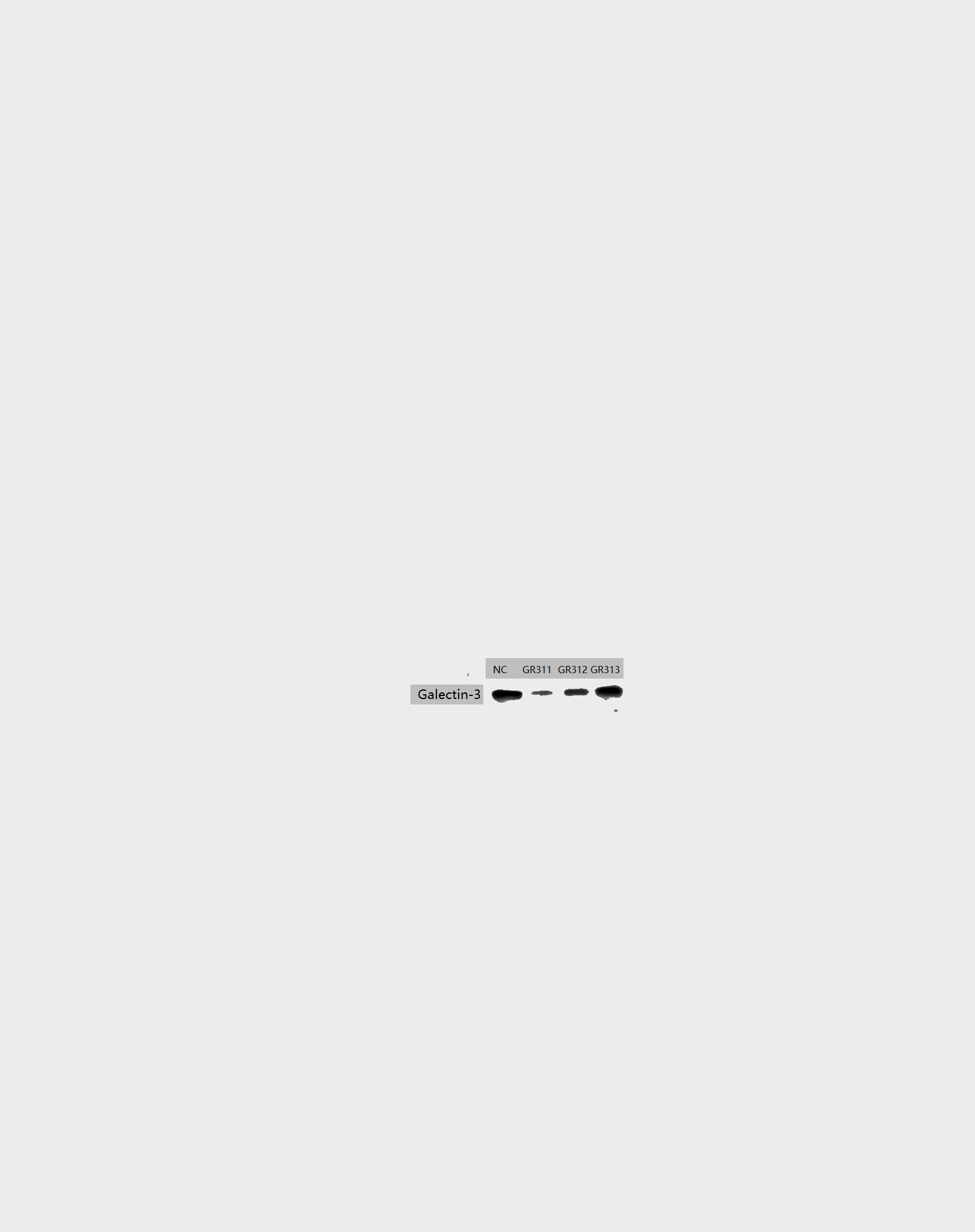

Supplement: S8 File — “Fig 4” and “Fig 4-GAPDH” files with corresponding suffixes present data from the same experiment. (ZIP) [file pone.0232166.s008.zip › Figure 4-1.tif]

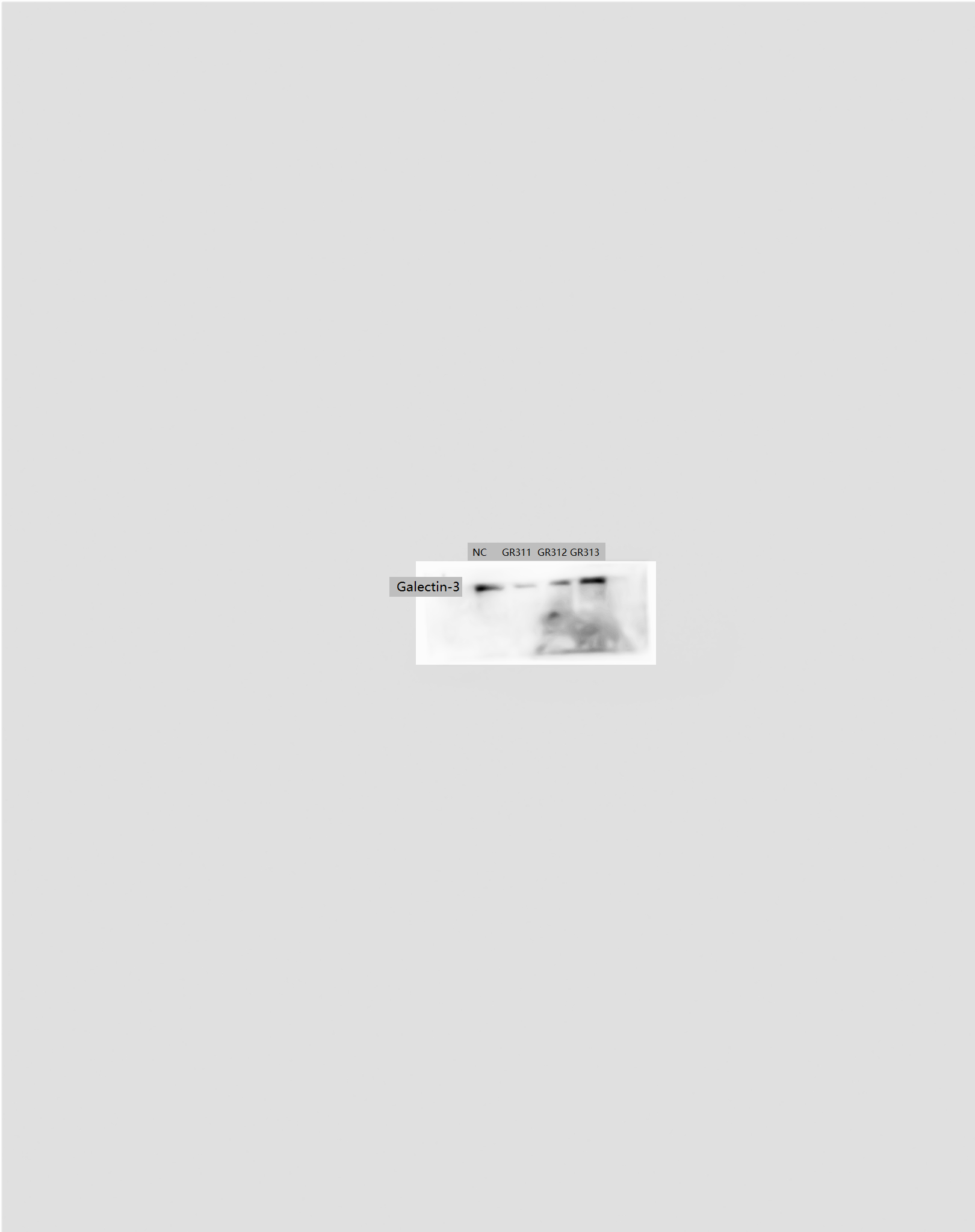

Supplement: S8 File — “Fig 4” and “Fig 4-GAPDH” files with corresponding suffixes present data from the same experiment. (ZIP) [file pone.0232166.s008.zip › Figure 4-2.tif]

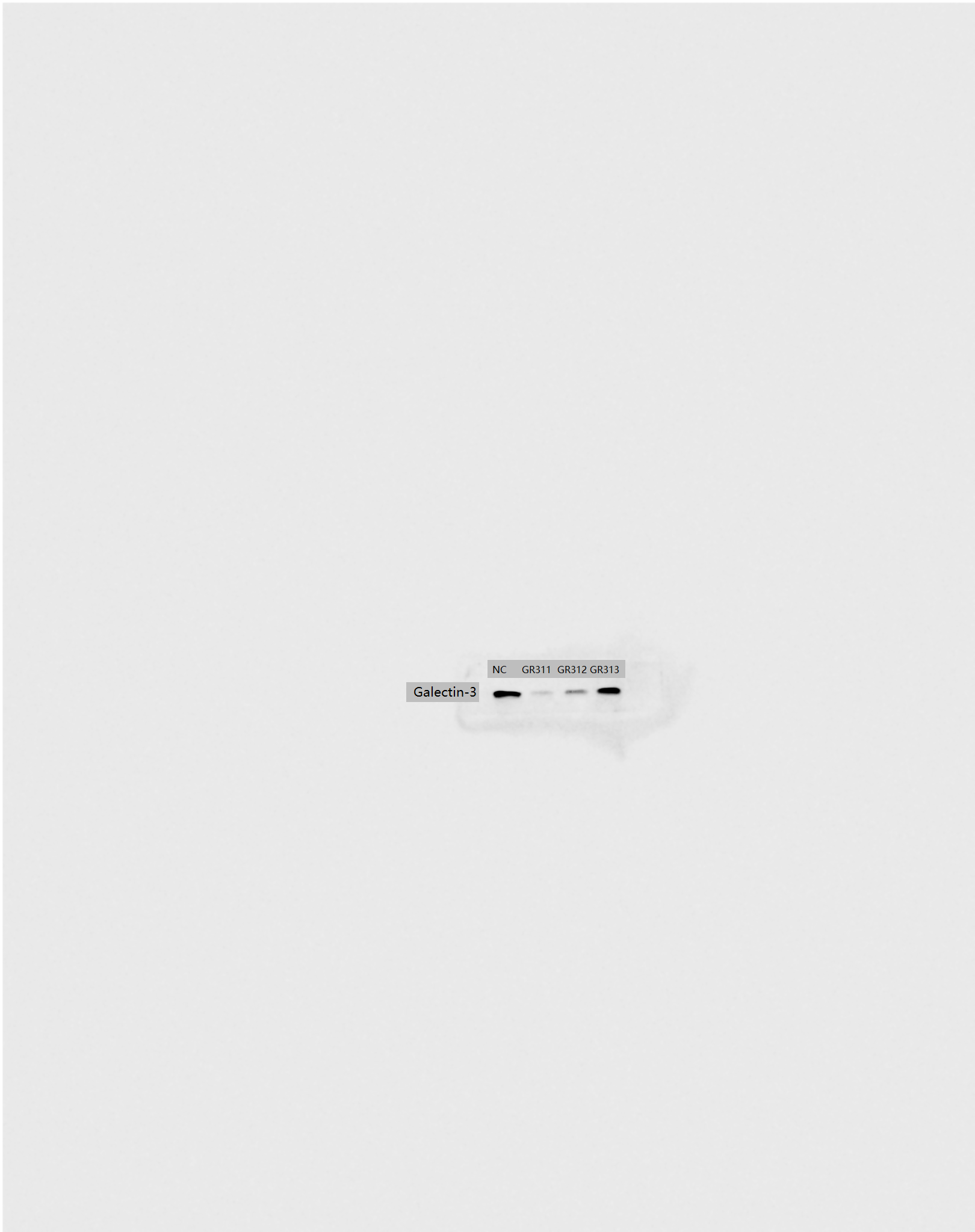

Supplement: S8 File — “Fig 4” and “Fig 4-GAPDH” files with corresponding suffixes present data from the same experiment. (ZIP) [file pone.0232166.s008.zip › Figure 4-3.tif]

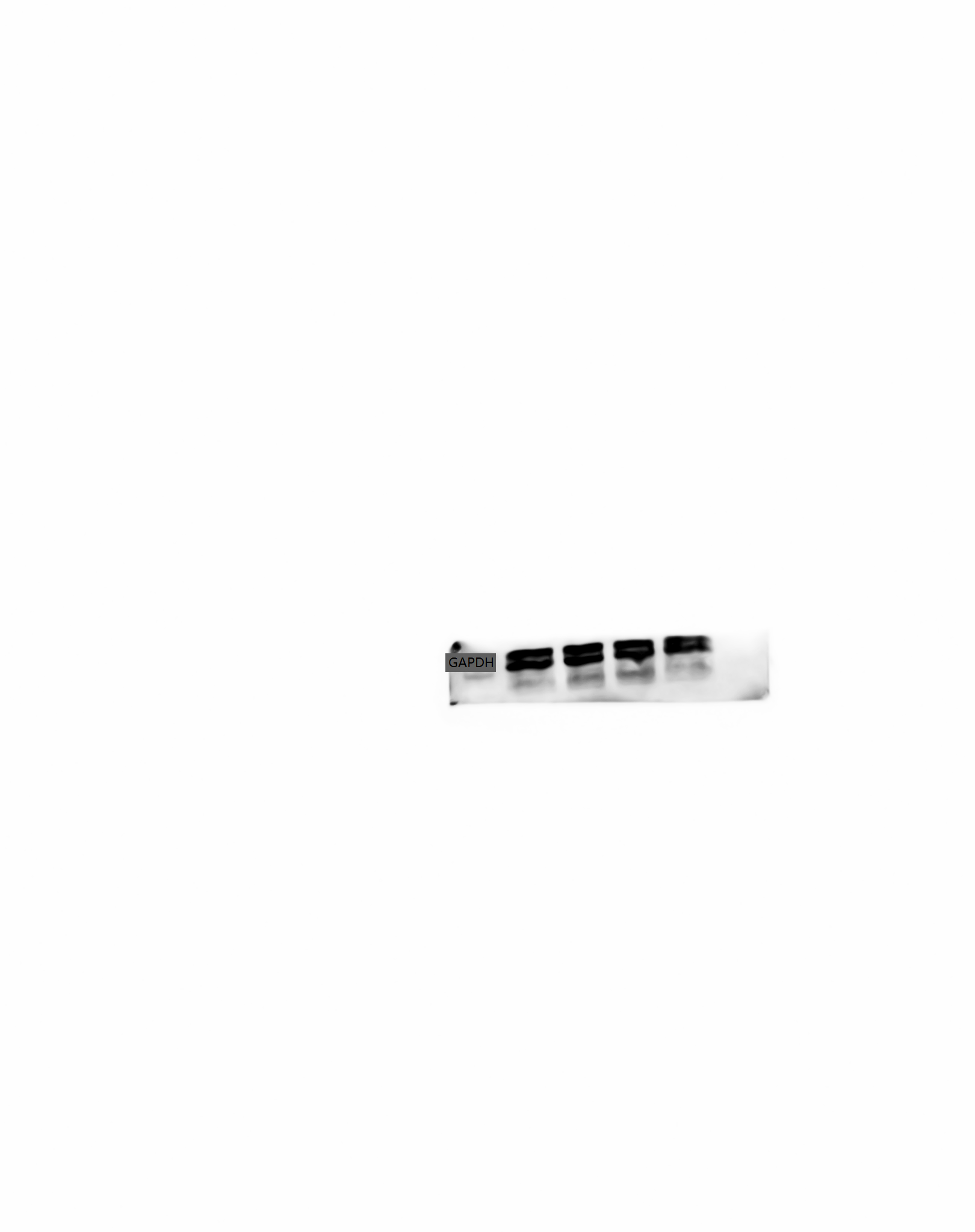

Supplement: S8 File — “Fig 4” and “Fig 4-GAPDH” files with corresponding suffixes present data from the same experiment. (ZIP) [file pone.0232166.s008.zip › Figure 4-GAPDH-1.tif]

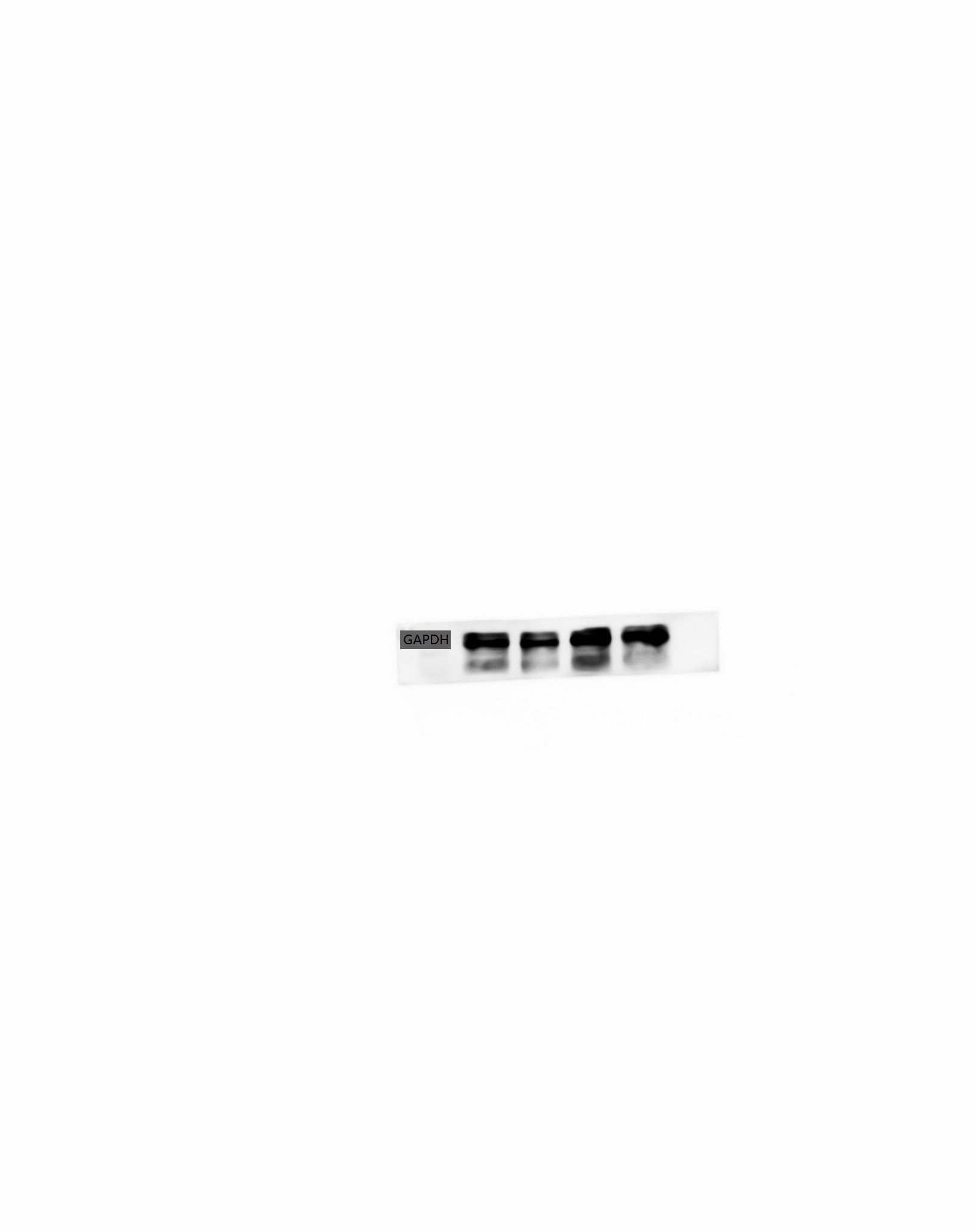

Supplement: S8 File — “Fig 4” and “Fig 4-GAPDH” files with corresponding suffixes present data from the same experiment. (ZIP) [file pone.0232166.s008.zip › Figure 4-GAPDH-2.tif]

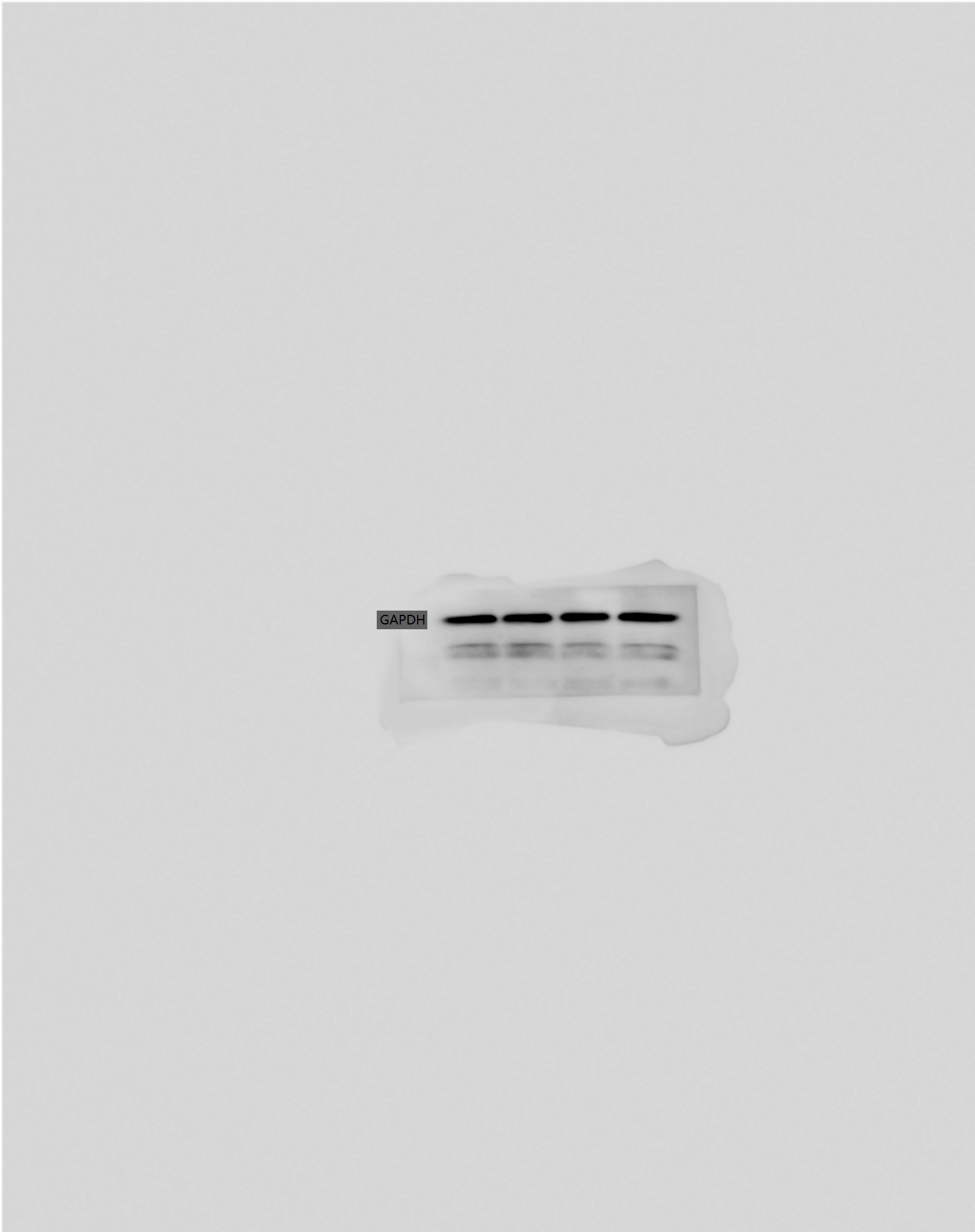

Supplement: S8 File — “Fig 4” and “Fig 4-GAPDH” files with corresponding suffixes present data from the same experiment. (ZIP) [file pone.0232166.s008.zip › Figure 4-GAPDH-3.tif]

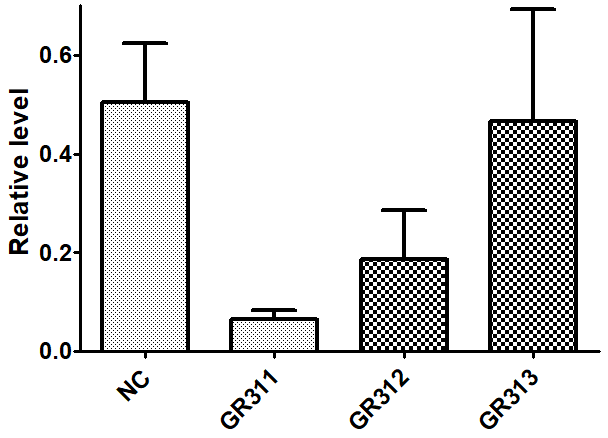

Supplement: S8 File — “Fig 4” and “Fig 4-GAPDH” files with corresponding suffixes present data from the same experiment. (ZIP) [file pone.0232166.s008.zip › Figure 4.tif]
