# Supplementary material for: Correction: Galectin-3 as a Marker and Potential Therapeutic Target in Breast Cancer
Source: PLoS One. 2020 Apr 16;15(4):e0232166. doi: 10.1371/journal.pone.0232166 (PMC7162513; doi:10.1371/journal.pone.0232166)
Supplement: S2 File — (PDF) [file pone.0232166.s002.pdf]

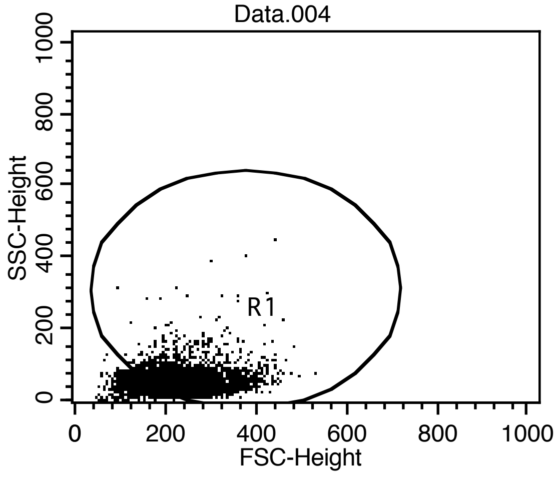


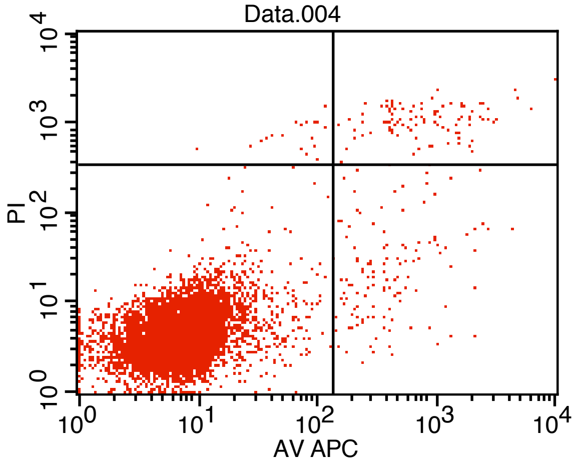


| Quad | Events | % Gated | % Total | X Mean | Y Mean |
|---|---|---|---|---|---|
| UL | 18 | 0.23 | 0.18 | 71.67 | 778.66 |
| UR | 103 | 1.29 | 1.03 | 1198.90 | 1155.36 |
| LL | 7728 | 96.95 | 77.28 | 8.80 | 5.53 |
| LR | 122 | 1.53 | 1.22 | 581.28 | 46.44 |

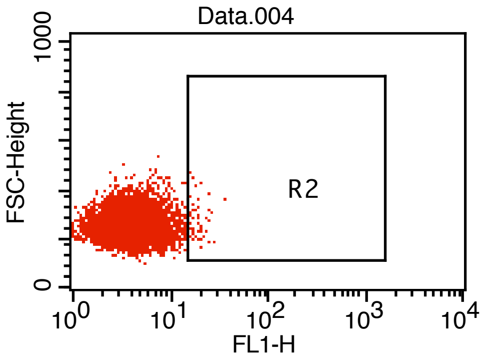


| Region | Events | % Gated | % Total |
|---|---|---|---|
| R1 | 7971 | 100.00 | 79.71 |
| R2 | 47 | 0.59 | 0.47 |

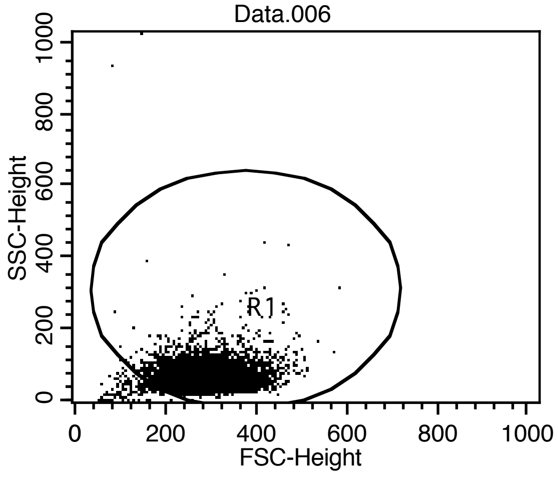


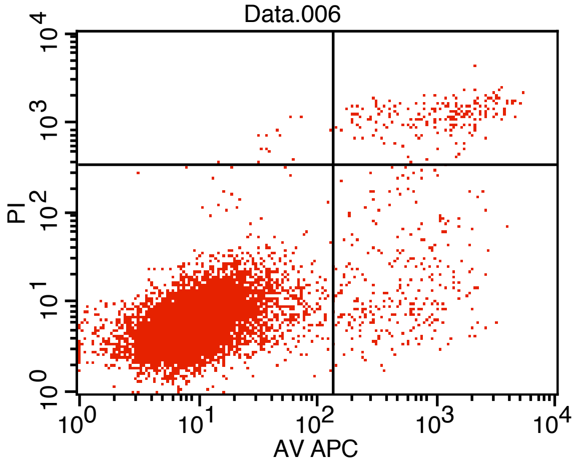


| Quad | Events | % Gated | % Total | X Mean | Y Mean |
|---|---|---|---|---|---|
| UL | 13 | 0.13 | 0.13 | 49.94 | 685.02 |
| UR | 203 | 2.08 | 2.03 | 1475.73 | 1295.54 |
| LL | 9267 | 94.93 | 92.67 | 12.45 | 7.42 |
| LR | 279 | 2.86 | 2.79 | 714.38 | 38.86 |

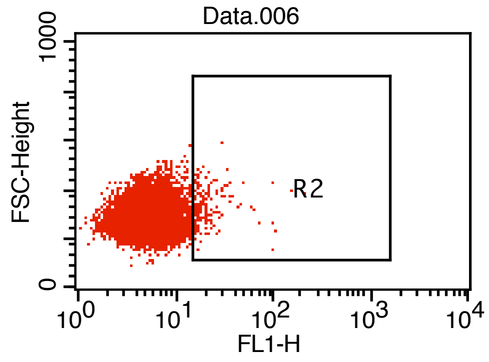


| Region | Events | % Gated | % Total |
|---|---|---|---|
| R1 | 9762 | 100.00 | 97.62 |
| R2 | 177 | 1.81 | 1.77 |

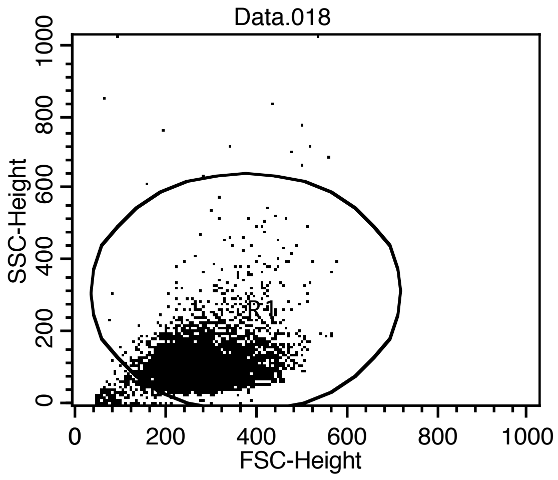


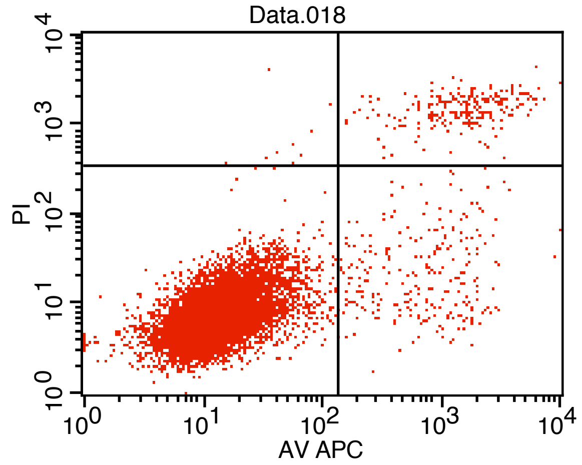


| Quad | Events | % Gated | % Total | X Mean | Y Mean |
|---|---|---|---|---|---|
| UL | 9 | 0.09 | 0.09 | 55.19 | 1025.66 |
| UR | 234 | 2.40 | 2.34 | 1959.80 | 1562.42 |
| LL | 9261 | 94.88 | 92.61 | 16.18 | 8.72 |
| LR | 257 | 2.63 | 2.57 | 997.12 | 37.15 |

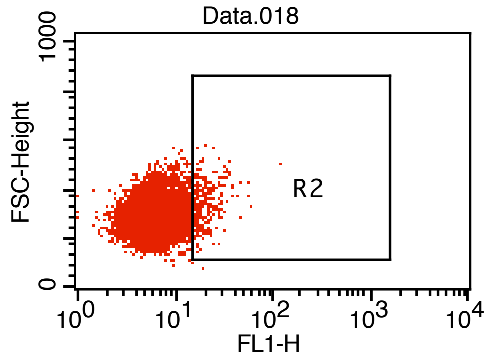


| Region | Events | % Gated | % Total |
|---|---|---|---|
| R1 | 9761 | 100.00 | 97.61 |
| R2 | 330 | 3.38 | 3.30 |

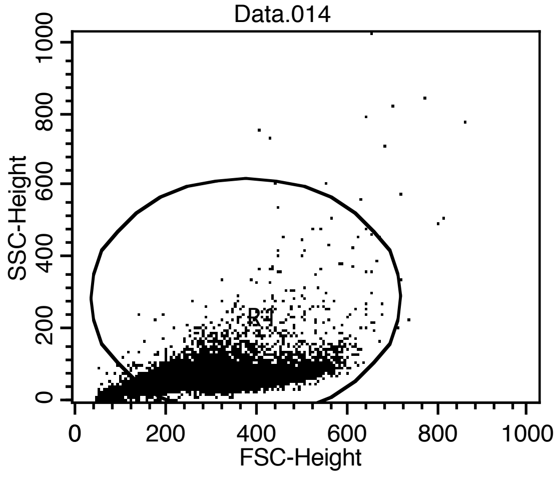


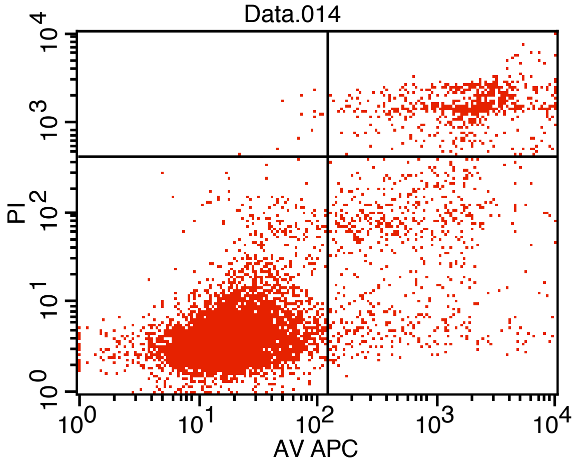


| Quad | Events | % Gated | % Total | X Mean | Y Mean |
|---|---|---|---|---|---|
| UL | 15 | 0.16 | 0.15 | 78.39 | 1197.76 |
| UR | 747 | 8.12 | 7.47 | 2670.12 | 1735.53 |
| LL | 7775 | 84.46 | 77.75 | 23.59 | 6.36 |
| LR | 668 | 7.26 | 6.68 | 1087.08 | 78.14 |

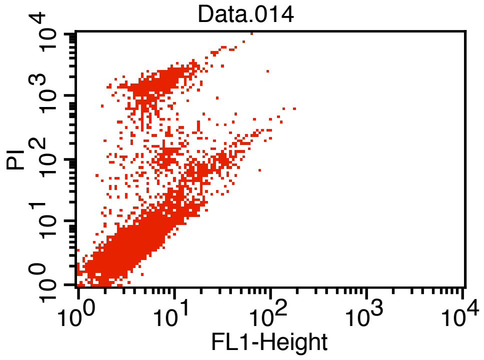


| Region | Events | % Gated | % Total |
|---|---|---|---|
| R1 | 9205 | 100.00 | 92.05 |
| R2 | 344 | 3.74 | 3.44 |

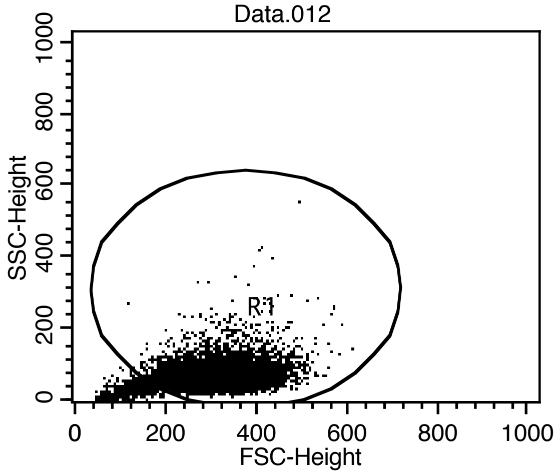


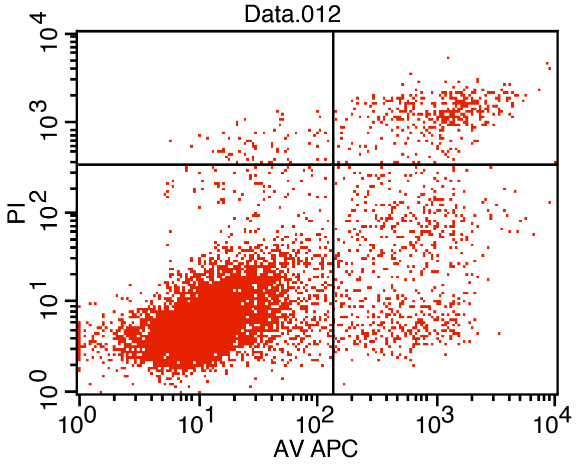


| Quad | Events | % Gated | % Total | X Mean | Y Mean |
|---|---|---|---|---|---|
| UL | 71 | 0.76 | 0.71 | 46.25 | 657.05 |
| UR | 411 | 4.37 | 4.11 | 1460.85 | 1388.15 |
| LL | 8238 | 87.63 | 82.38 | 16.31 | 9.50 |
| LR | 681 | 7.24 | 6.81 | 782.54 | 55.68 |

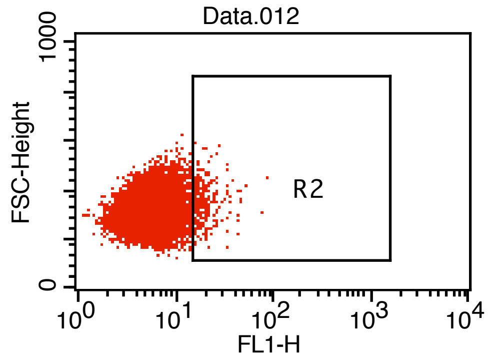


| Region | Events | % Gated | % Total |
|---|---|---|---|
| R1 | 9401 | 100.00 | 94.01 |
| R2 | 259 | 2.76 | 2.59 |

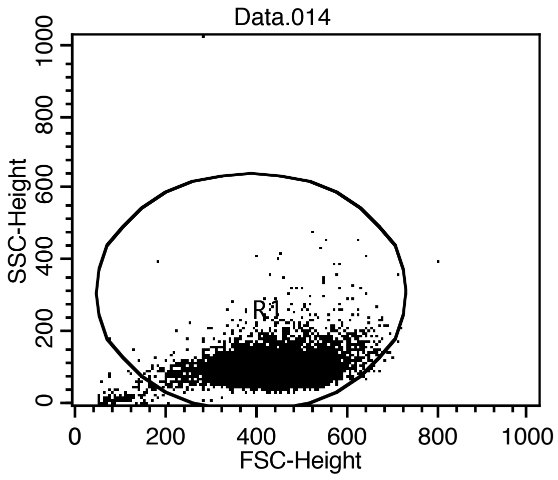


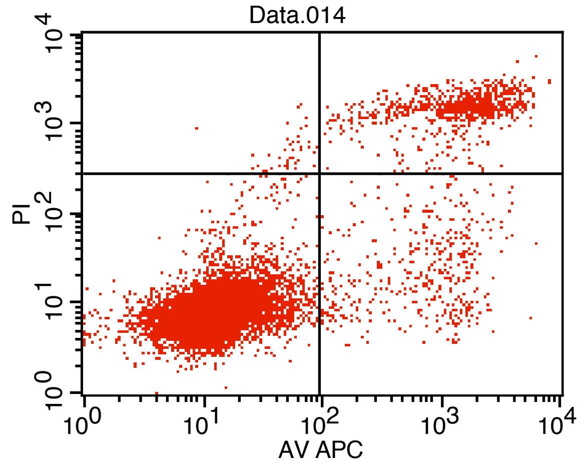


| Quad | Events | % Gated | % Total | X Mean | Y Mean |
|---|---|---|---|---|---|
| UL | 47 | 0.48 | 0.47 | 54.68 | 559.28 |
| UR | 857 | 8.69 | 8.57 | 1848.10 | 1594.81 |
| LL | 8536 | 86.51 | 85.36 | 15.36 | 9.86 |
| LR | 427 | 4.33 | 4.27 | 959.53 | 39.16 |

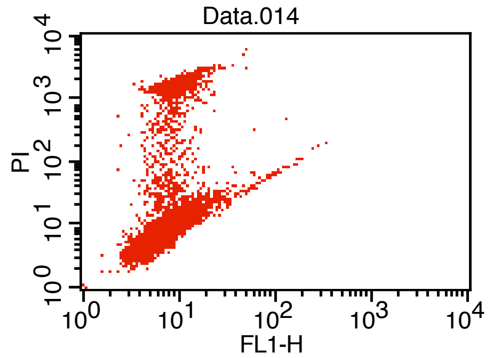


| Region | Events | % Gated | % Total |
|---|---|---|---|
| R1 | 9867 | 100.00 | 98.67 |
| R2 | 444 | 4.50 | 4.44 |

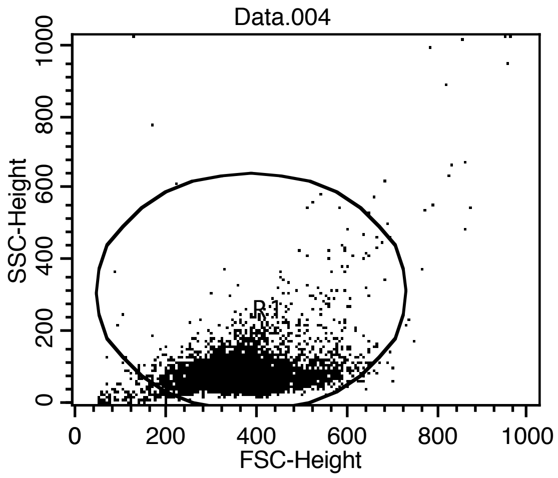


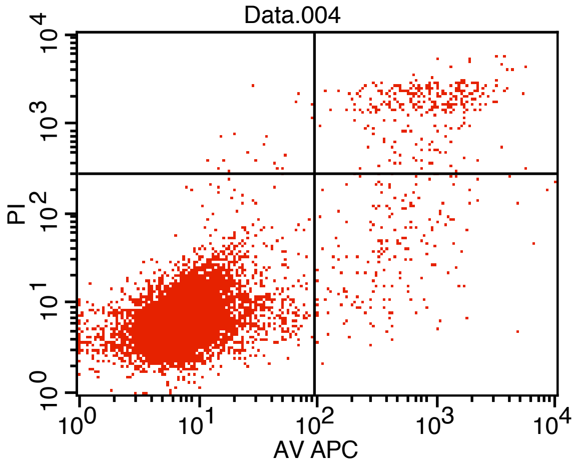


| Quad | Events | % Gated | % Total | X Mean | Y Mean |
|---|---|---|---|---|---|
| UL | 19 | 0.19 | 0.19 | 40.22 | 844.85 |
| UR | 314 | 3.20 | 3.14 | 1113.32 | 1864.59 |
| LL | 9280 | 94.70 | 92.80 | 9.47 | 8.33 |
| LR | 186 | 1.90 | 1.86 | 727.53 | 67.02 |

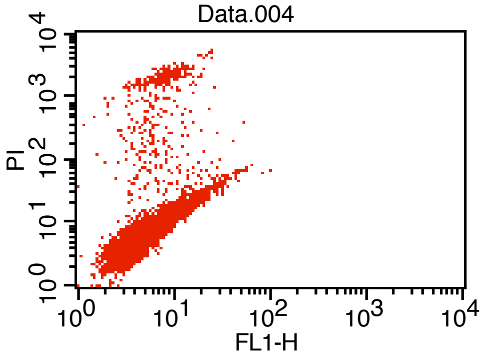


| Region | Events | % Gated | % Total |
|---|---|---|---|
| R1 | 9799 | 100.00 | 97.99 |
| R2 | 287 | 2.93 | 2.87 |

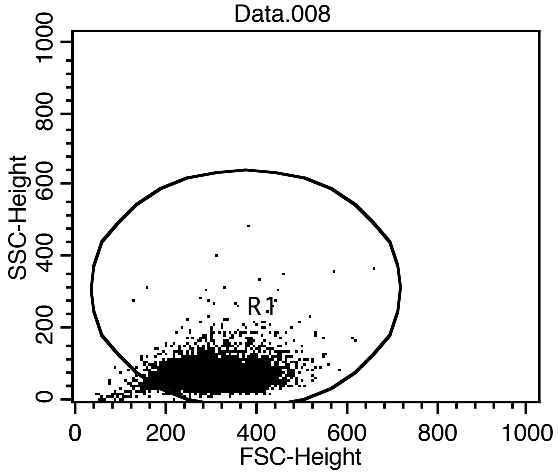


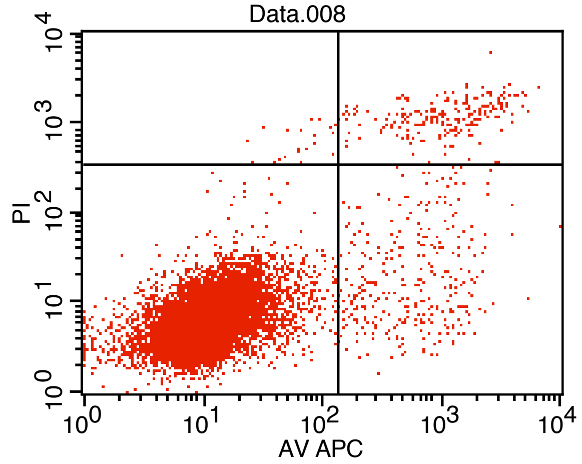


| Quad | Events | % Gated | % Total | X Mean | Y Mean |
|---|---|---|---|---|---|
| UL | 23 | 0.23 | 0.23 | 76.66 | 673.45 |
| UR | 215 | 2.18 | 2.15 | 1502.52 | 1322.74 |
| LL | 9301 | 94.42 | 93.01 | 13.93 | 7.91 |
| LR | 312 | 3.17 | 3.12 | 801.97 | 42.79 |

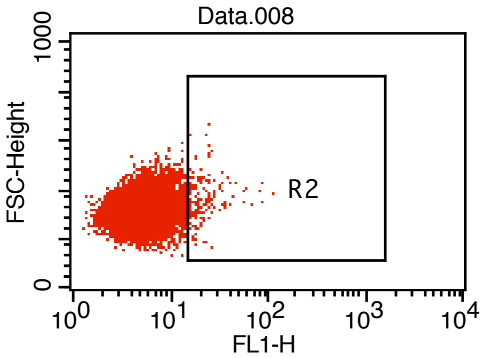


| Region | Events | % Gated | % Total |
|---|---|---|---|
| R1 | 9851 | 100.00 | 98.51 |
| R2 | 184 | 1.87 | 1.84 |

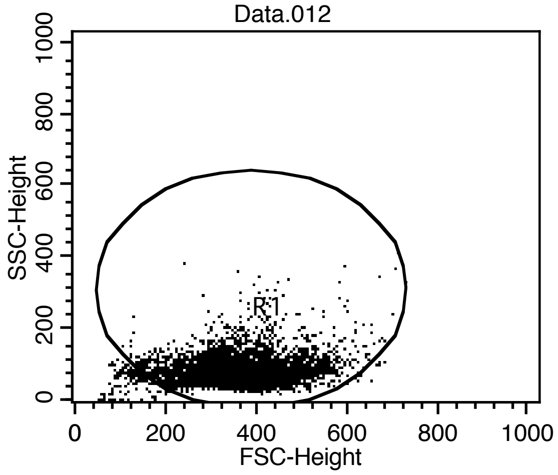


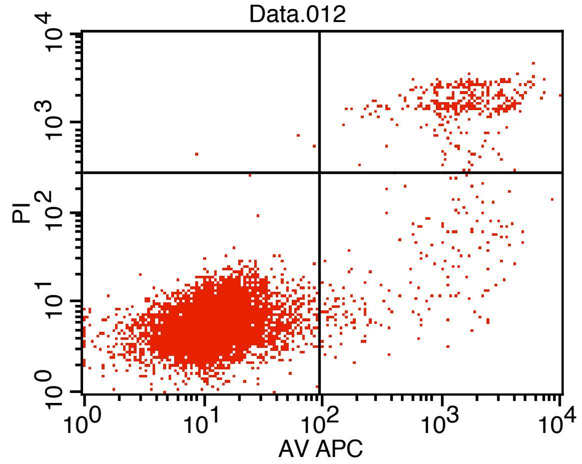


| Quad | Events | % Gated | % Total | X Mean | Y Mean |
|---|---|---|---|---|---|
| UL | 3 | 0.03 | 0.03 | 52.63 | 568.23 |
| UR | 352 | 3.58 | 3.52 | 1957.79 | 1768.88 |
| LL | 9299 | 94.51 | 92.99 | 14.06 | 6.34 |
| LR | 185 | 1.88 | 1.85 | 1058.81 | 40.08 |

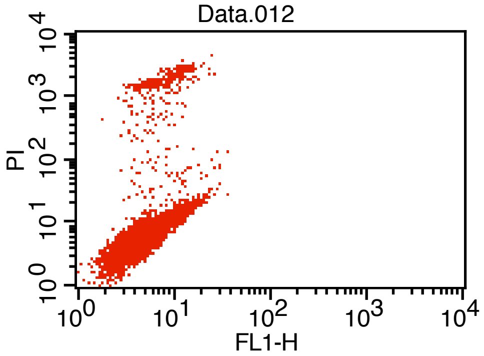


| Region | Events | % Gated | % Total |
|---|---|---|---|
| R1 | 9839 | 100.00 | 98.39 |
| R2 | 111 | 1.13 | 1.11 |

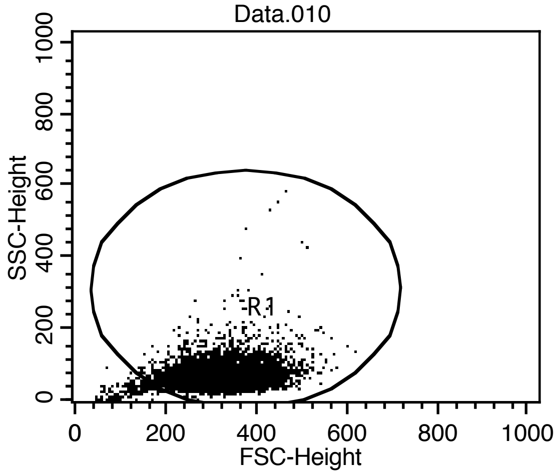


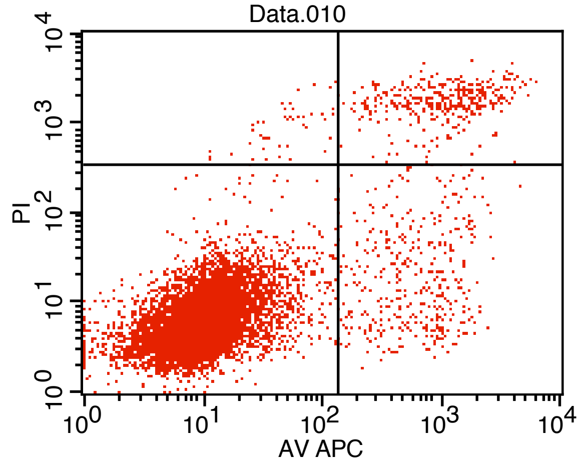


| Quad | Events | % Gated | % Total | X Mean | Y Mean |
|---|---|---|---|---|---|
| UL | 46 | 0.47 | 0.46 | 55.02 | 1041.02 |
| UR | 385 | 3.93 | 3.85 | 1424.56 | 1900.07 |
| LL | 8989 | 91.67 | 89.89 | 12.53 | 8.03 |
| LR | 386 | 3.94 | 3.86 | 771.19 | 42.64 |

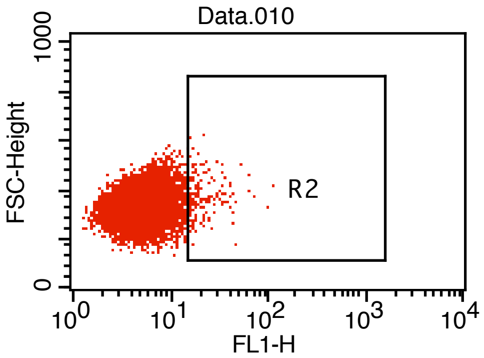


| Region | Events | % Gated | % Total |
|---|---|---|---|
| R1 | 9806 | 100.00 | 98.06 |
| R2 | 172 | 1.75 | 1.72 |

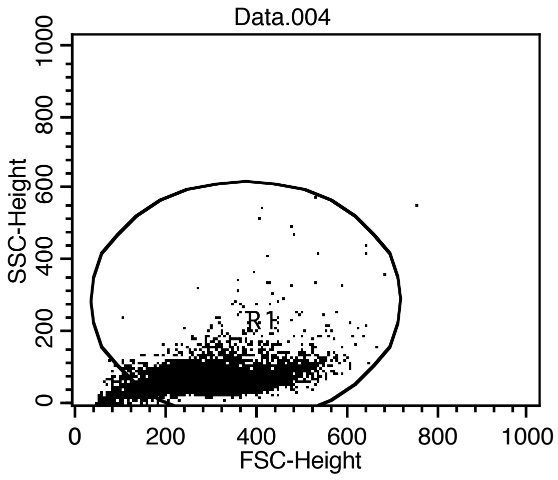


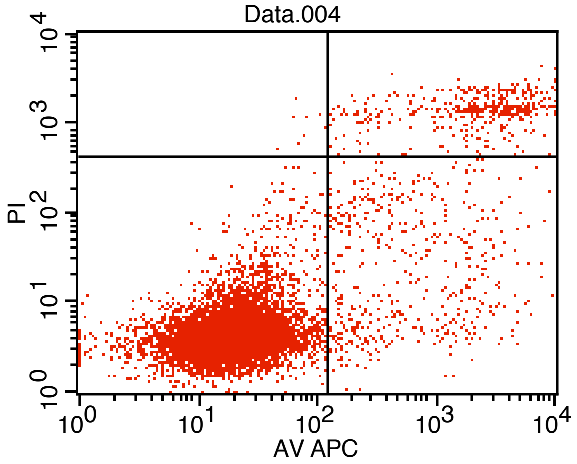


| Quad | Events | % Gated | % Total | X Mean | Y Mean |
|---|---|---|---|---|---|
| UL | 8 | 0.08 | 0.08 | 77.41 | 1023.83 |
| UR | 451 | 4.75 | 4.51 | 3048.28 | 1501.66 |
| LL | 8523 | 89.83 | 85.23 | 22.55 | 6.05 |
| LR | 506 | 5.33 | 5.06 | 1060.82 | 57.35 |

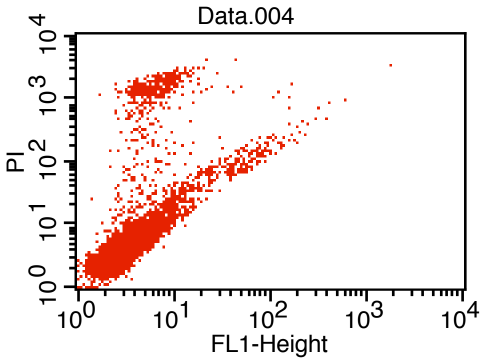


| Region | Events | % Gated | % Total |
|---|---|---|---|
| R1 | 9488 | 100.00 | 94.88 |
| R2 | 249 | 2.62 | 2.49 |

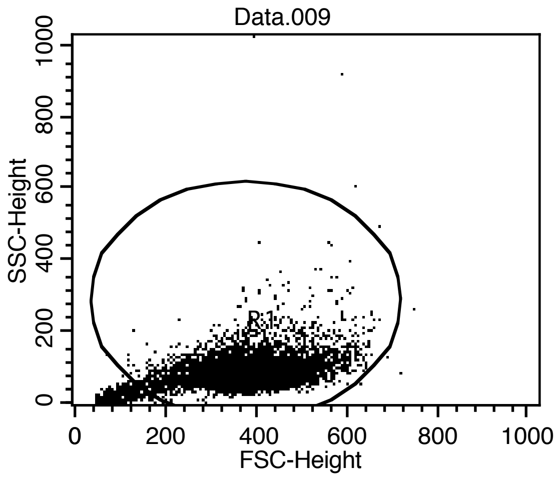


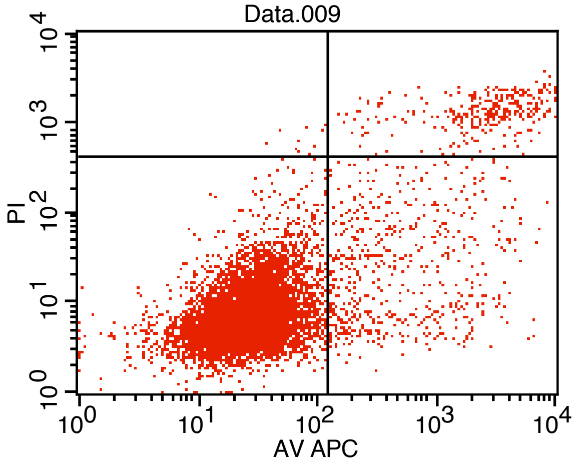


| Quad | Events | % Gated | % Total | X Mean | Y Mean |
|---|---|---|---|---|---|
| UL | 11 | 0.11 | 0.11 | 76.20 | 659.03 |
| UR | 346 | 3.61 | 3.46 | 3554.39 | 1448.70 |
| LL | 8605 | 89.90 | 86.05 | 30.13 | 9.83 |
| LR | 610 | 6.37 | 6.10 | 862.49 | 54.74 |

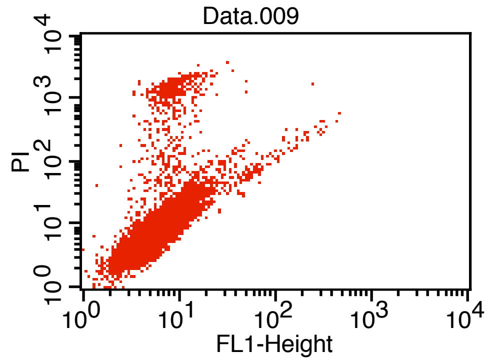


| Region | Events | % Gated | % Total |
|---|---|---|---|
| R1 | 9572 | 100.00 | 95.72 |
| R2 | 400 | 4.18 | 4.00 |
