# Supplementary material for: Correction: Galectin-3 as a Marker and Potential Therapeutic Target in Breast Cancer
Source: PLoS One. 2020 Apr 16;15(4):e0232166. doi: 10.1371/journal.pone.0232166 (PMC7162513; doi:10.1371/journal.pone.0232166)
Supplement: S5 File — (PDF) [file pone.0232166.s005.pdf]

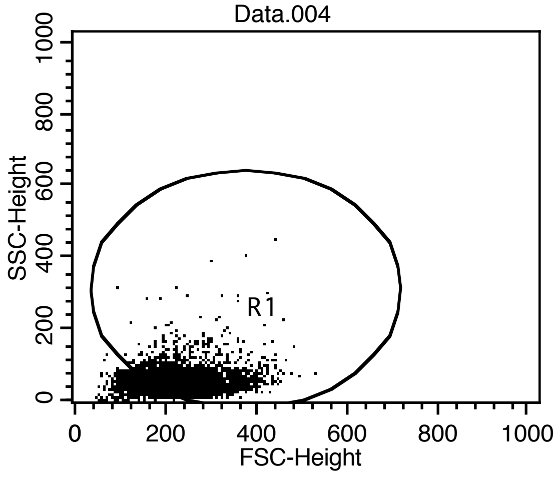

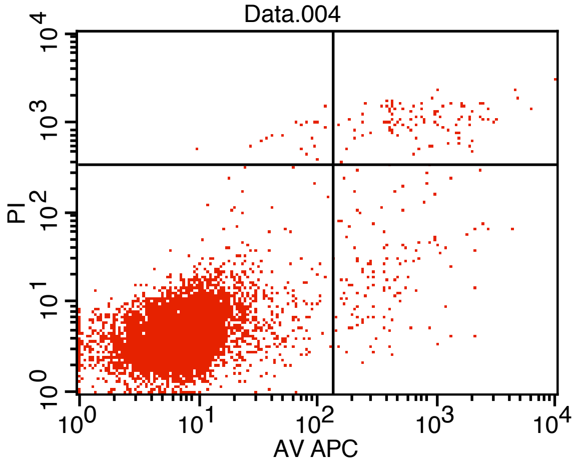

Quad	Events	% Gated	% Total	X Mean	Y Mean
UL	18	0.23	0.18	71.67	778.66
UR	103	1.29	1.03	1198.90	1155.36
LL	7728	96.95	77.28	8.80	5.53
LR	122	1.53	1.22	581.28	46.44

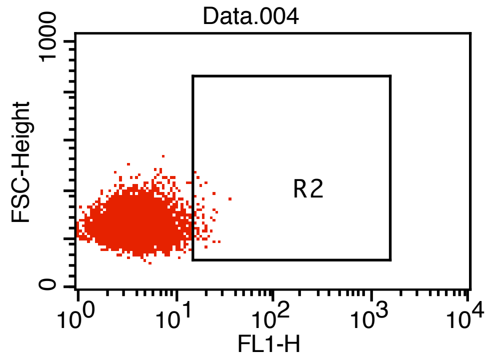

Region	Events	% Gated	% Total
R1	7971	100.00	79.71
R2	47	0.59	0.47

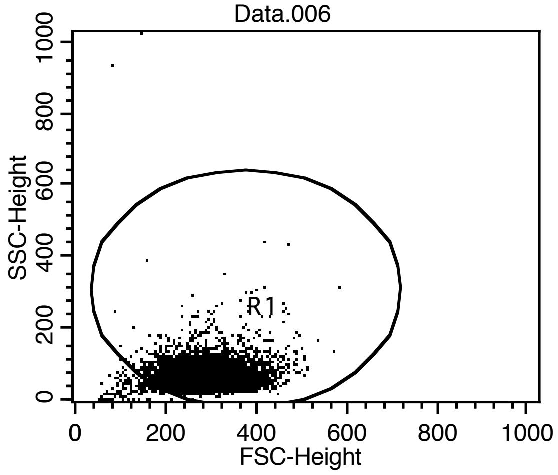

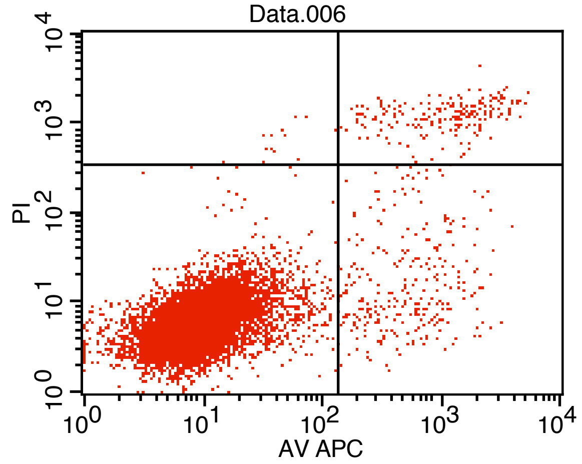

Quad	Events	% Gated	% Total	X Mean	Y Mean
UL	13	0.13	0.13	49.94	685.02
UR	203	2.08	2.03	1475.73	1295.54
LL	9267	94.93	92.67	12.45	7.42
LR	279	2.86	2.79	714.38	38.86

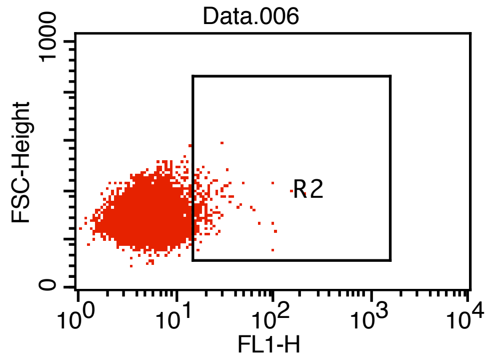

Region	Events	% Gated	% Total
R1	9762	100.00	97.62
R2	177	1.81	1.77

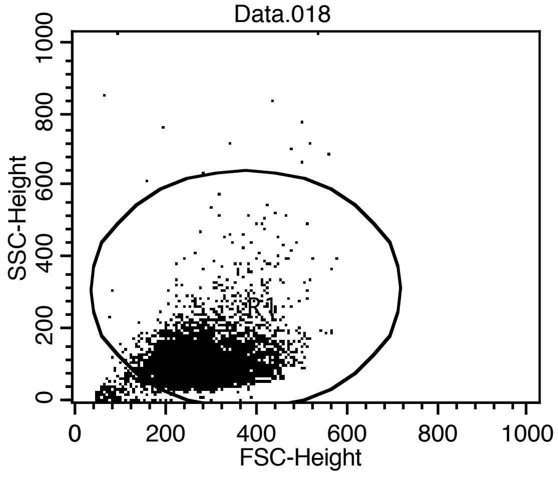

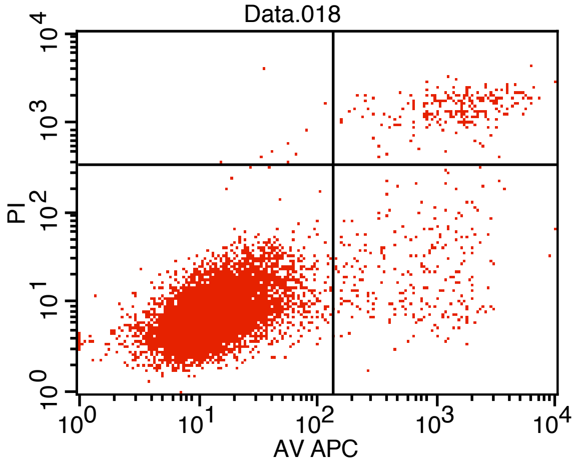

Quad	Events	% Gated	% Total	X Mean	Y Mean
UL	9	0.09	0.09	55.19	1025.66
UR	234	2.40	2.34	1959.80	1562.42
LL	9261	94.88	92.61	16.18	8.72
LR	257	2.63	2.57	997.12	37.15

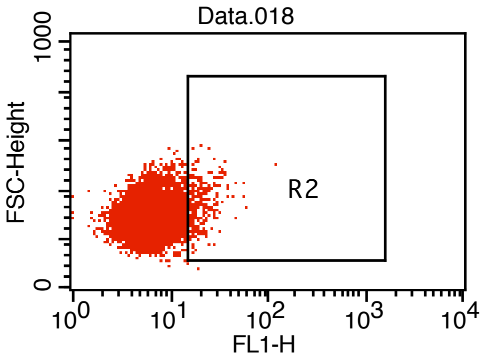

Region	Events	% Gated	% Total
R1	9761	100.00	97.61
R2	330	3.38	3.30

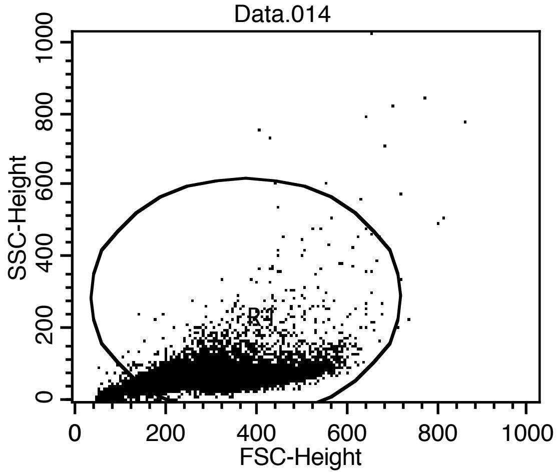

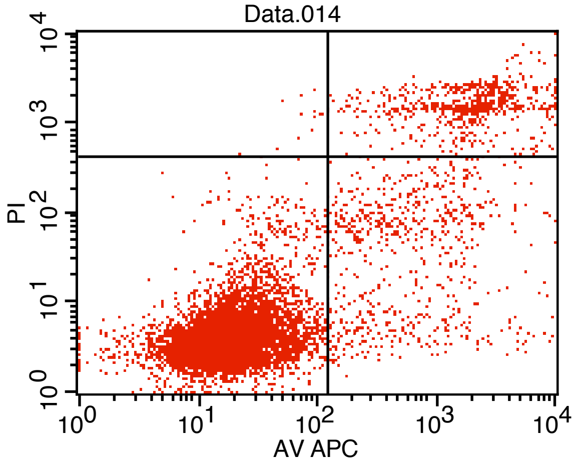

Quad	Events	% Gated	% Total	X Mean	Y Mean
UL	15	0.16	0.15	78.39	1197.76
UR	747	8.12	7.47	2670.12	1735.53
LL	7775	84.46	77.75	23.59	6.36
LR	668	7.26	6.68	1087.08	78.14

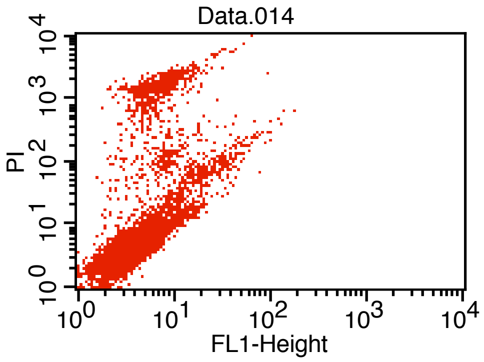

Region	Events	% Gated	% Total
R1	9205	100.00	92.05
R2	344	3.74	3.44

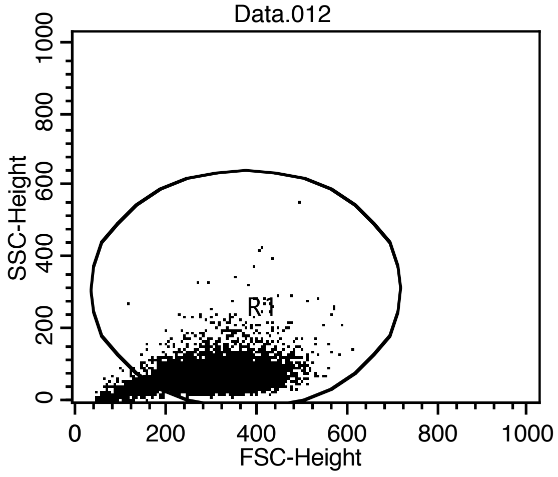

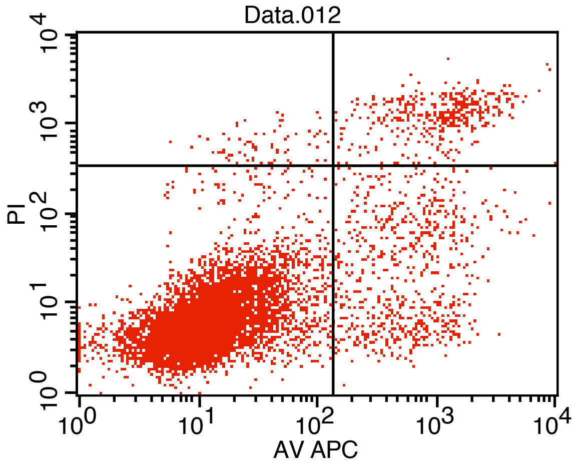

Quad	Events	% Gated	% Total	X Mean	Y Mean
UL	71	0.76	0.71	46.25	657.05
UR	411	4.37	4.11	1460.85	1388.15
LL	8238	87.63	82.38	16.31	9.50
LR	681	7.24	6.81	782.54	55.68

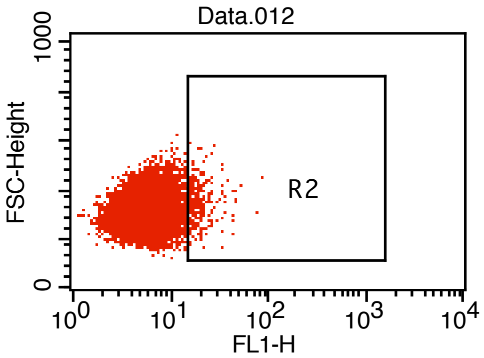

Region	Events	% Gated	% Total
R1	9401	100.00	94.01
R2	259	2.76	2.59

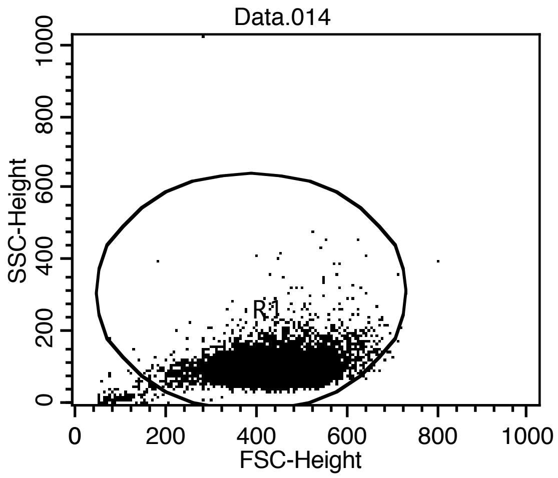

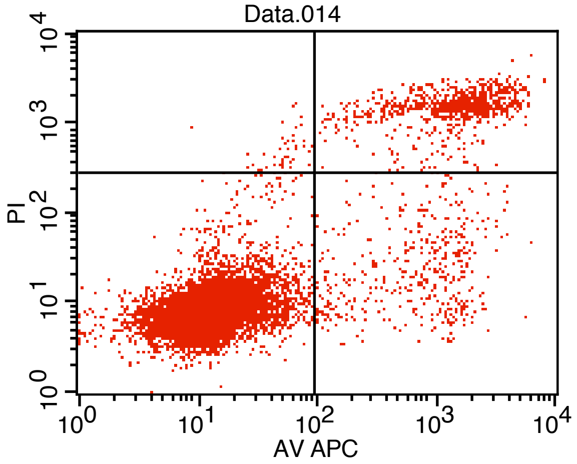

Quad	Events	% Gated	% Total	X Mean	Y Mean
UL	47	0.48	0.47	54.68	559.28
UR	857	8.69	8.57	1848.10	1594.81
LL	8536	86.51	85.36	15.36	9.86
LR	427	4.33	4.27	959.53	39.16

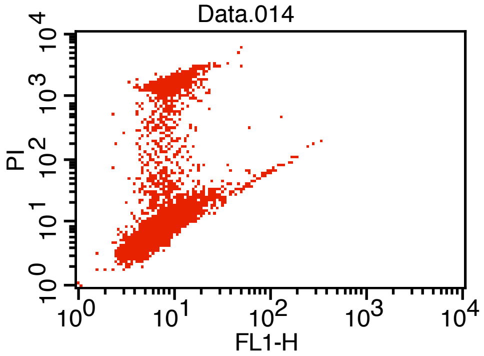

Region	Events	% Gated	% Total
R1	9867	100.00	98.67
R2	444	4.50	4.44

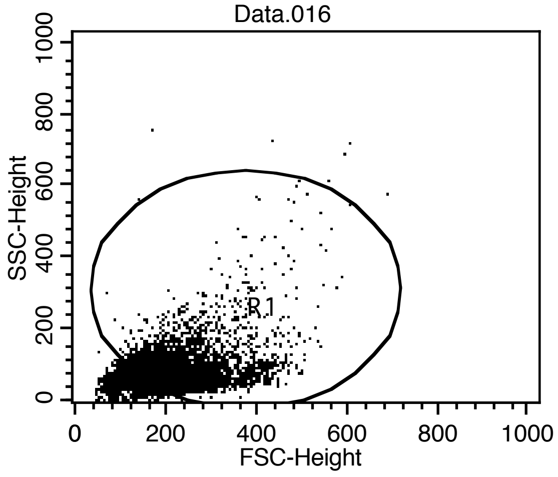

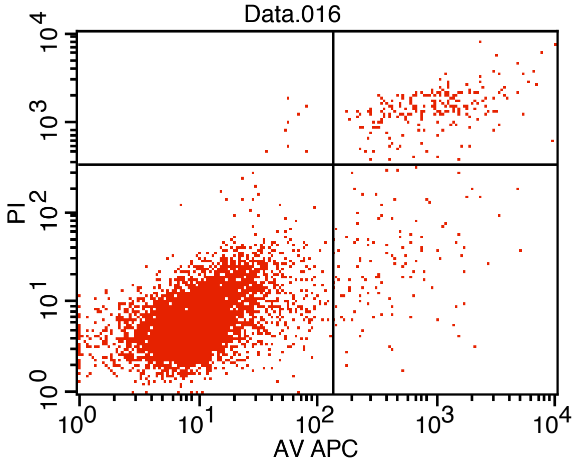

Quad	Events	% Gated	% Total	X Mean	Y Mean
UL	9	0.13	0.09	60.66	1014.72
UR	216	3.02	2.16	1429.02	1678.57
LL	6807	95.18	68.07	12.31	7.91
LR	120	1.68	1.20	676.78	51.28

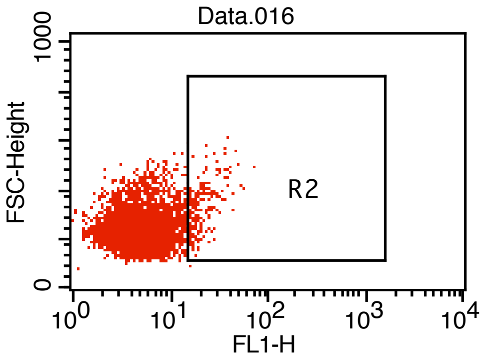

Region	Events	% Gated	% Total
R1	7152	100.00	71.52
R2	219	3.06	2.19

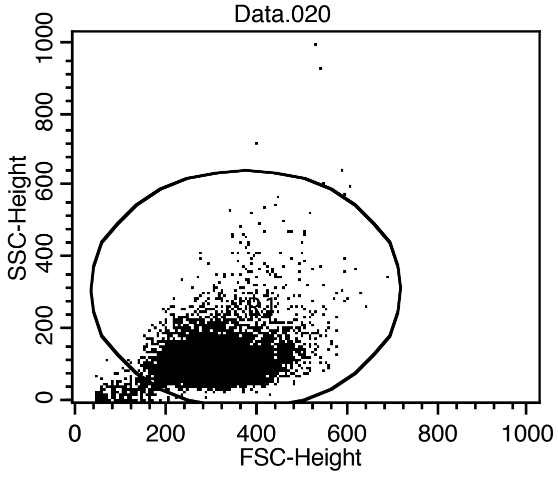

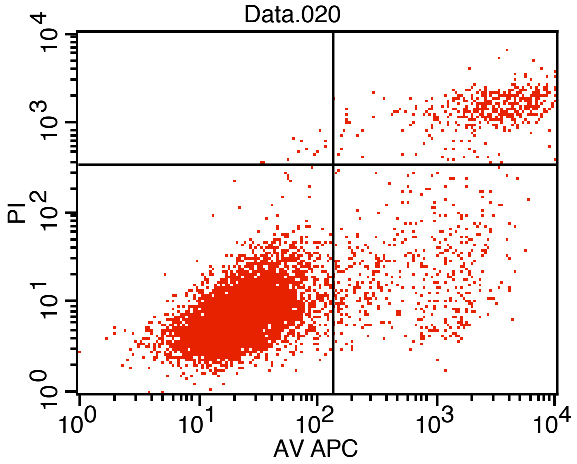

Quad	Events	% Gated	% Total	X Mean	Y Mean
UL	13	0.13	0.13	82.47	637.75
UR	434	4.45	4.34	3668.86	1582.01
LL	8822	90.44	88.22	24.89	8.38
LR	486	4.98	4.86	1044.62	37.42

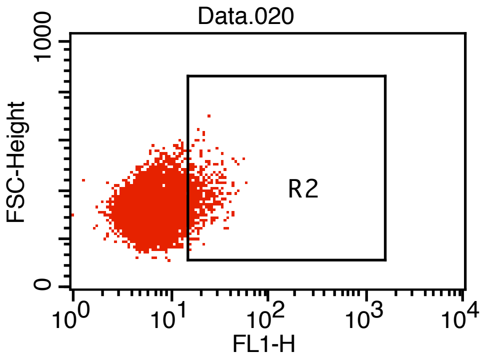

Region	Events	% Gated	% Total
R1	9755	100.00	97.55
R2	442	4.53	4.42

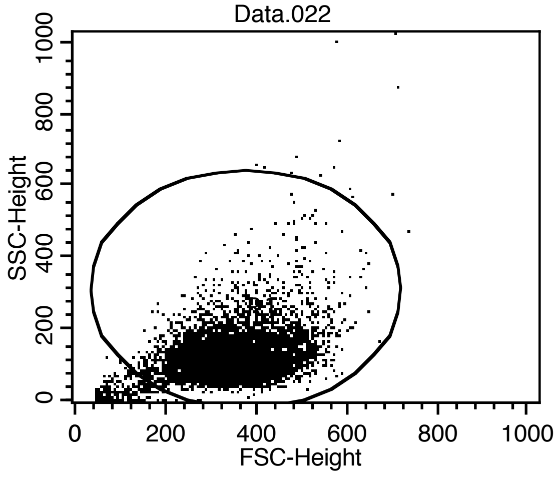

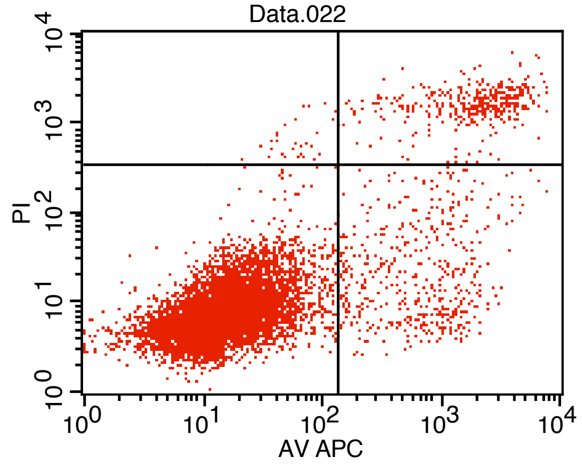

Quad	Events	% Gated	% Total	X Mean	Y Mean
UL	30	0.31	0.30	64.66	724.88
UR	379	3.89	3.79	2390.67	1745.78
LL	8827	90.69	88.27	17.84	9.11
LR	497	5.11	4.97	924.16	44.27

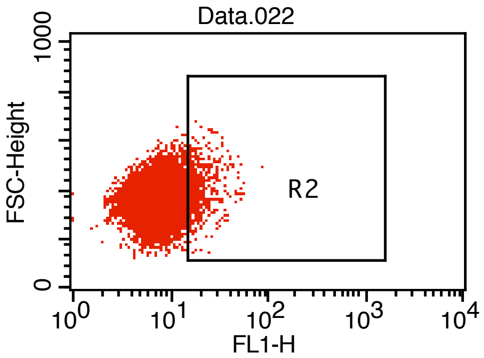

Region	Events	% Gated	% Total
R1	9733	100.00	97.33
R2	581	5.97	5.81

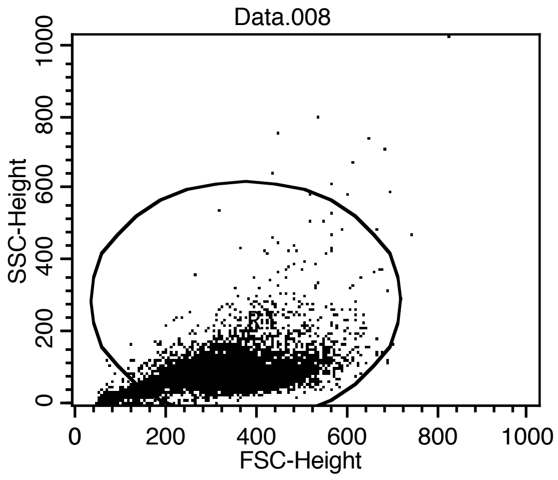

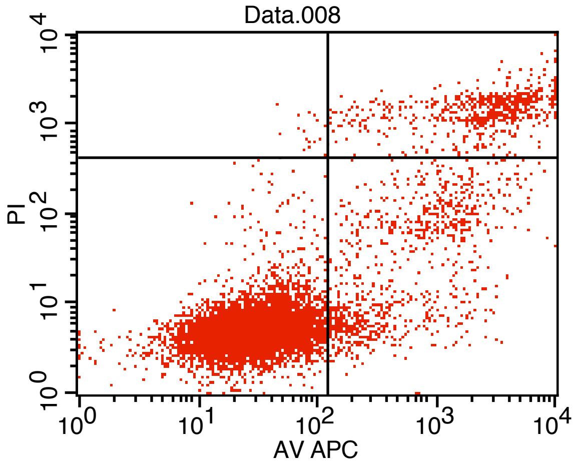

Quad	Events	% Gated	% Total	X Mean	Y Mean
UL	20	0.21	0.20	85.77	856.03
UR	663	7.00	6.63	3489.07	1574.75
LL	7860	82.99	78.60	33.89	6.32
LR	928	9.80	9.28	908.01	61.45

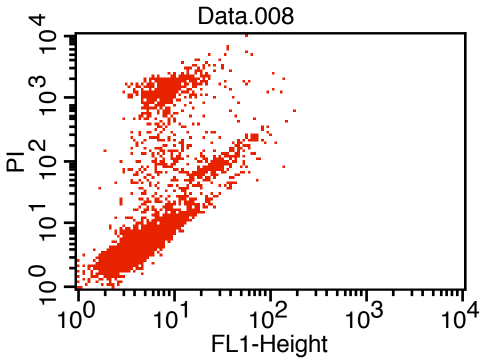

Region	Events	% Gated	% Total
R1	9471	100.00	94.71
R2	411	4.34	4.11

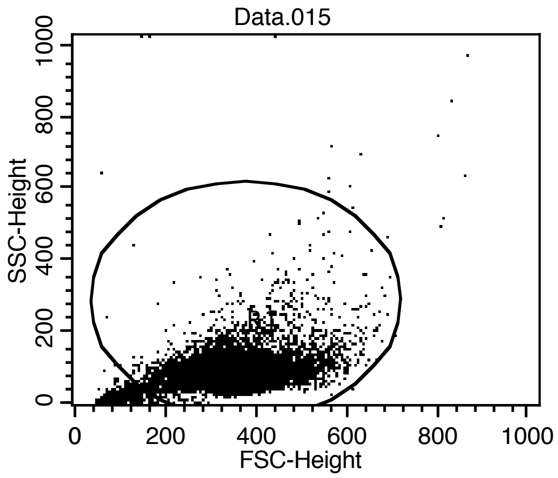

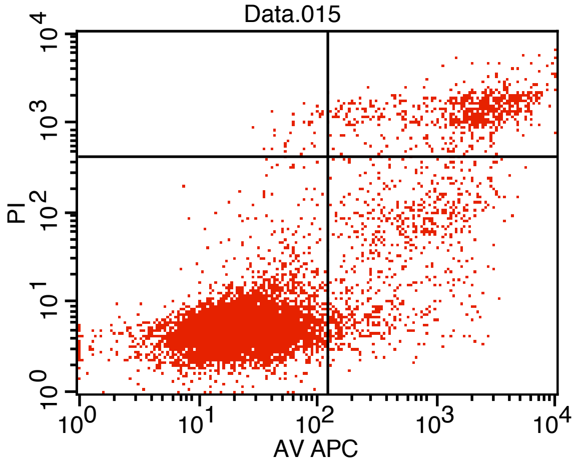

Quad	Events	% Gated	% Total	X Mean	Y Mean
UL	33	0.35	0.33	73.97	924.77
UR	684	7.24	6.84	2595.30	1536.04
LL	7953	84.24	79.53	27.71	6.44
LR	771	8.17	7.71	743.46	72.24

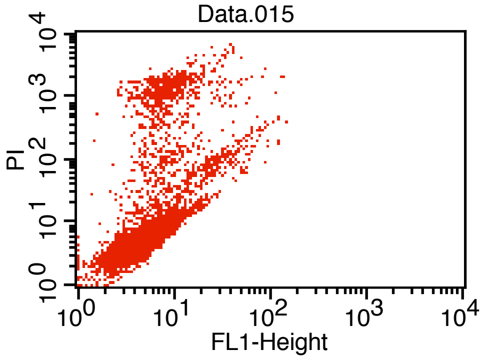

Region	Events	% Gated	% Total
R1	9441	100.00	94.41
R2	409	4.33	4.09

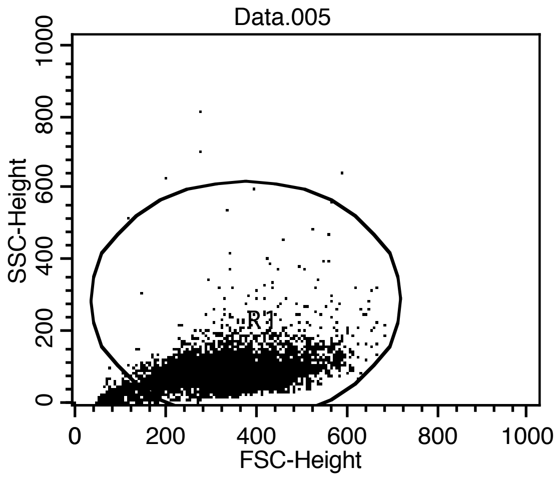

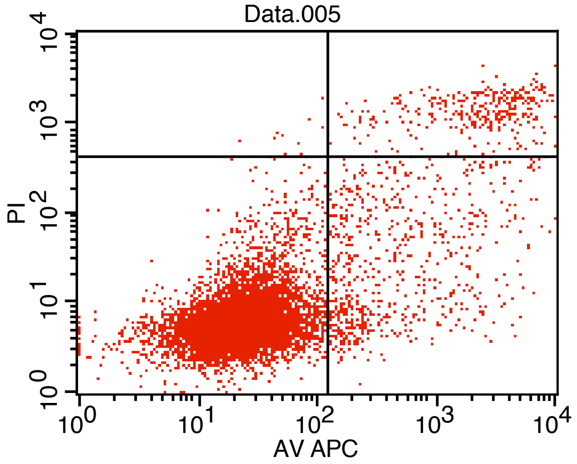

Quad	Events	% Gated	% Total	X Mean	Y Mean
UL	10	0.11	0.10	66.25	828.13
UR	329	3.46	3.29	3078.33	1482.50
LL	8485	89.14	84.85	27.65	8.58
LR	695	7.30	6.95	919.48	68.70

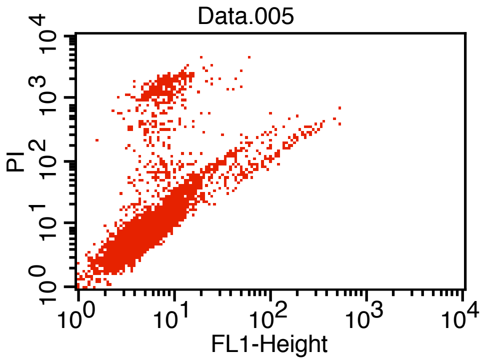

Region	Events	% Gated	% Total
R1	9519	100.00	95.19
R2	392	4.12	3.92

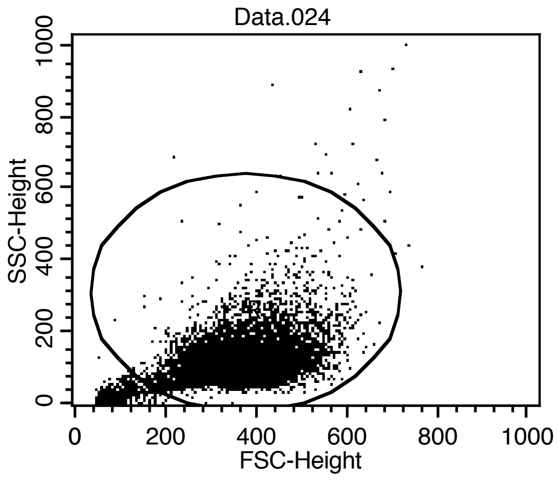

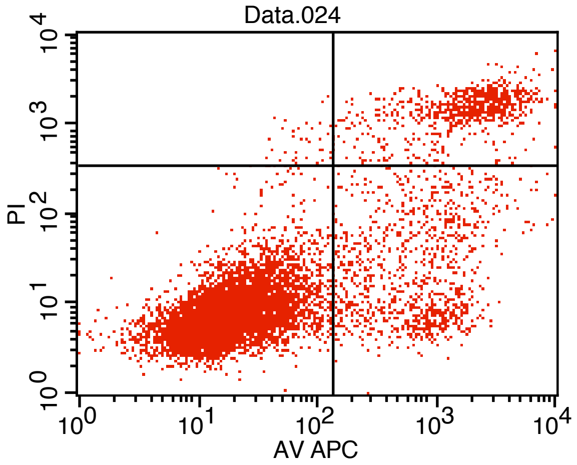

Quad	Events	% Gated	% Total	X Mean	Y Mean
UL	41	0.43	0.41	81.17	683.52
UR	776	8.12	7.76	2427.78	1570.62
LL	7762	81.24	77.62	23.12	9.69
LR	975	10.21	9.75	990.20	52.46

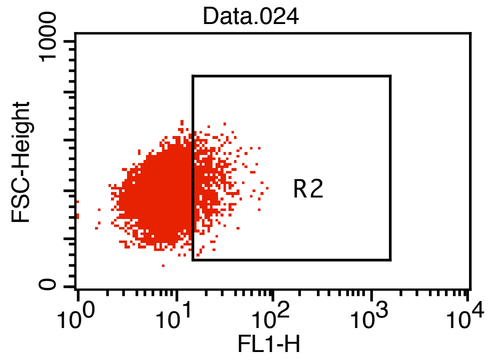

Region	Events	% Gated	% Total
R1	9554	100.00	95.54
R2	710	7.43	7.10

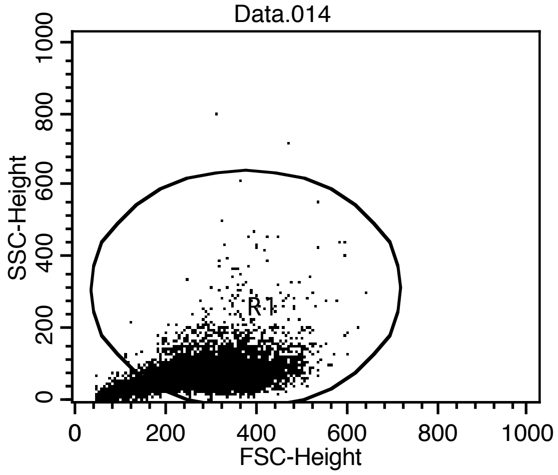

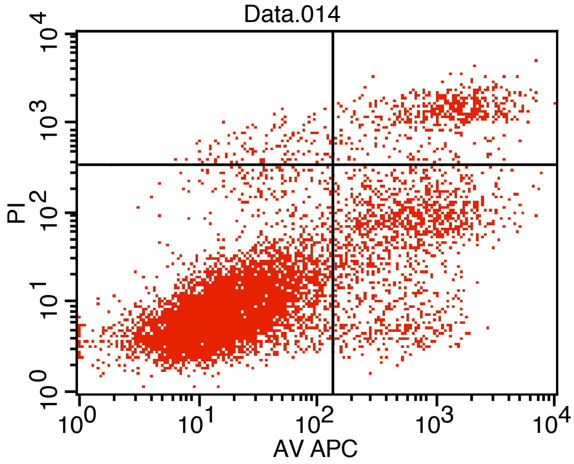

Quad	Events	% Gated	% Total	X Mean	Y Mean
UL	120	1.38	1.20	51.14	634.95
UR	530	6.10	5.30	1622.94	1390.75
LL	6737	77.52	67.37	23.47	13.92
LR	1304	15.00	13.04	791.79	76.86

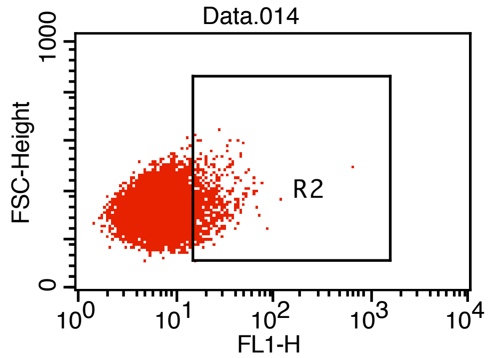

Region	Events	% Gated	% Total
R1	8691	100.00	86.91
R2	567	6.52	5.67

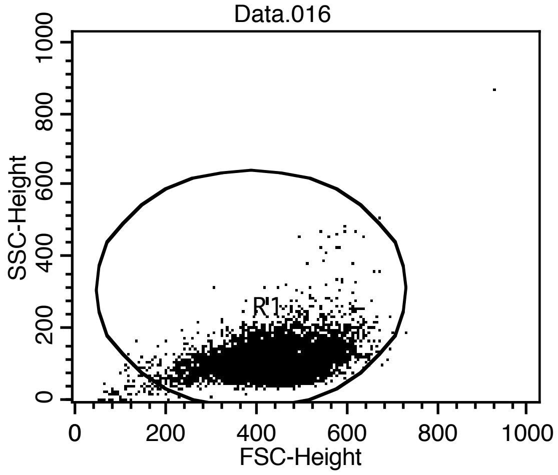

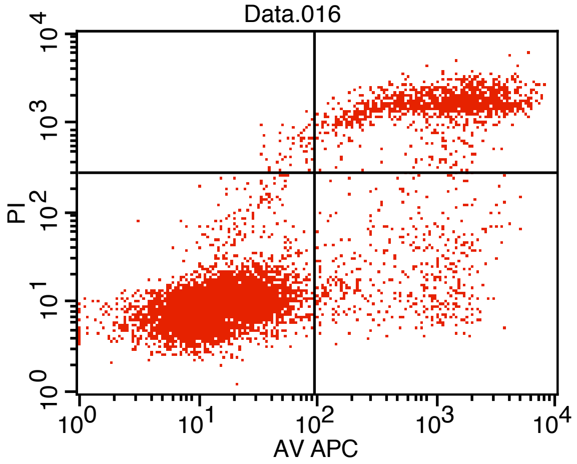

Quad	Events	% Gated	% Total	X Mean	Y Mean
UL	52	0.52	0.52	67.26	627.73
UR	1418	14.31	14.18	1706.19	1688.01
LL	7986	80.59	79.86	16.70	10.65
LR	453	4.57	4.53	915.14	37.68

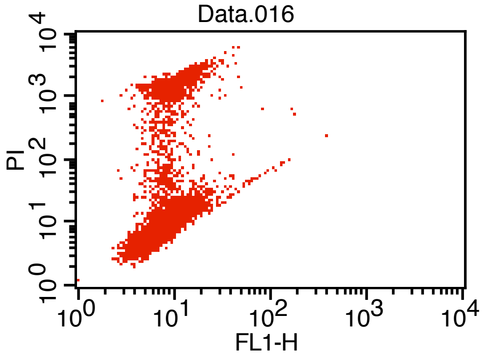

Region	Events	% Gated	% Total
R1	9909	100.00	99.09
R2	497	5.02	4.97

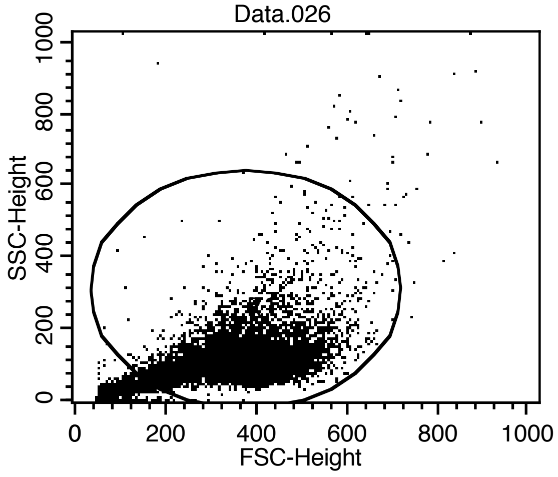

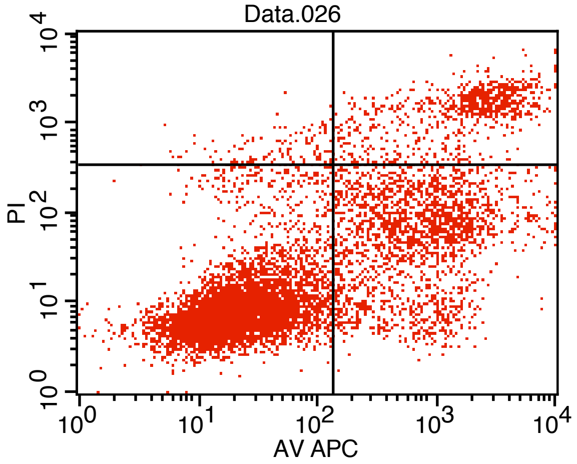

Quad	Events	% Gated	% Total	X Mean	Y Mean
UL	128	1.43	1.28	60.64	579.21
UR	795	8.90	7.95	2418.38	1574.51
LL	5963	66.75	59.63	27.80	16.48
LR	2047	22.92	20.47	1097.68	85.86

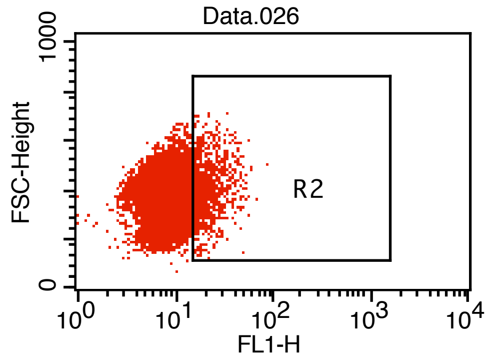

Region	Events	% Gated	% Total
R1	8933	100.00	89.33
R2	1071	11.99	10.71

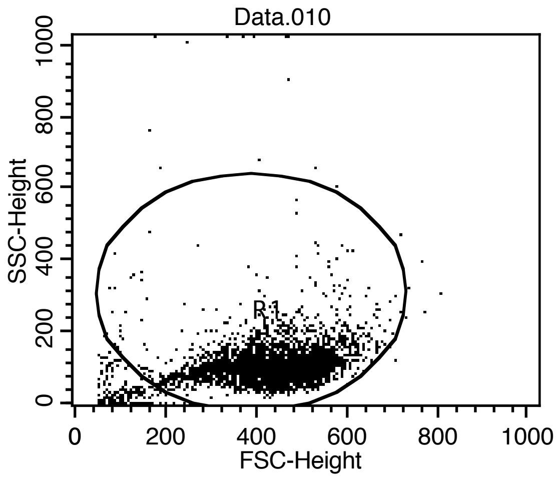

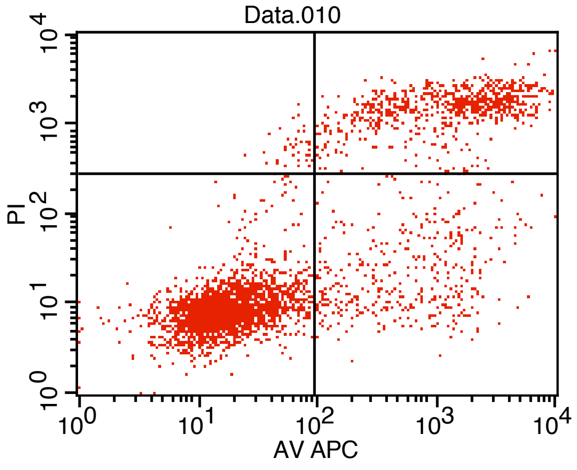

Quad	Events	% Gated	% Total	X Mean	Y Mean
UL	39	1.09	1.04	66.83	639.62
UR	768	21.52	20.40	2091.79	1695.93
LL	2368	66.37	62.90	21.85	10.93
LR	393	11.01	10.44	980.48	37.09

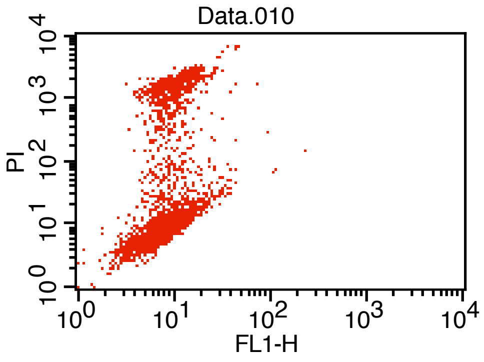

Region	Events	% Gated	% Total
R1	3568	100.00	94.77
R2	293	8.21	7.78

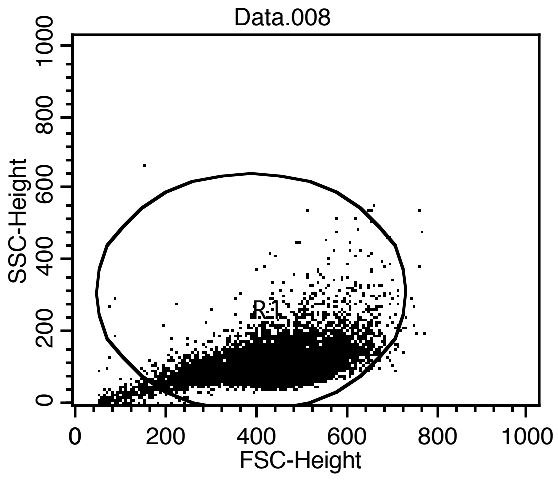

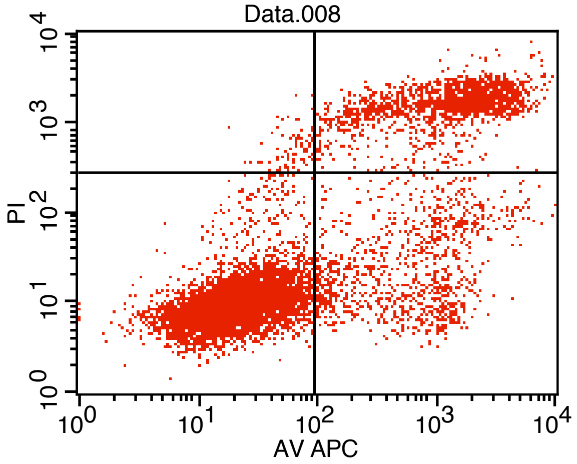

Quad	Events	% Gated	% Total	X Mean	Y Mean
UL	84	0.86	0.84	64.47	596.59
UR	1868	19.23	18.68	1924.96	1727.61
LL	6700	68.96	67.00	23.74	12.13
LR	1064	10.95	10.64	932.40	44.88

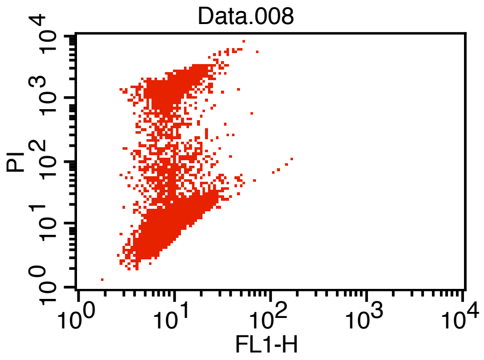

Region	Events	% Gated	% Total
R1	9716	100.00	97.16
R2	901	9.27	9.01
