# Supplementary material for: Correction: Galectin-3 as a Marker and Potential Therapeutic Target in Breast Cancer
Source: PLoS One. 2020 Apr 16;15(4):e0232166. doi: 10.1371/journal.pone.0232166 (PMC7162513; doi:10.1371/journal.pone.0232166)
Supplement: S12 File — (DOCX) [file pone.0232166.s012.docx]

| **Case Processing Summary** | | | | | | |
| --- | --- | --- | --- | --- | --- | --- |
|  | Cases | | | | | |
|  | Valid | | Missing | | Total | |
|  | N | Percent | N | Percent | N | Percent |
| Age * Galectin3status | 1187 | 100.0% | 0 | 0.0% | 1187 | 100.0% |
| Tumorstage * Galectin3status | 1187 | 100.0% | 0 | 0.0% | 1187 | 100.0% |
| Histologicalgrade * Galectin3status | 1187 | 100.0% | 0 | 0.0% | 1187 | 100.0% |
| Tumorsize * Galectin3status | 1187 | 100.0% | 0 | 0.0% | 1187 | 100.0% |
| Metastaticnodes * Galectin3status | 1187 | 100.0% | 0 | 0.0% | 1187 | 100.0% |
| Her2status * Galectin3status | 1187 | 100.0% | 0 | 0.0% | 1187 | 100.0% |
| Triplenegativebreastcancer * Galectin3status | 1187 | 100.0% | 0 | 0.0% | 1187 | 100.0% |

**Age * Galectin3status**

| **Crosstab** | | | | |
| --- | --- | --- | --- | --- |
| Count | | | | |
|  | | Galectin3status | | Total |
|  |  | Negative | Positive |  |
| Age | <35 Y | 139 | 89 | 228 |
|  | ≥35 Y | 660 | 299 | 959 |
| Total | | 799 | 388 | 1187 |

| **Chi-Square Tests** | | | | | |
| --- | --- | --- | --- | --- | --- |
|  | Value | df | Asymptotic Significance (2-sided) | Exact Sig. (2-sided) | Exact Sig. (1-sided) |
| Pearson Chi-Square | 5.168^a^ | 1 | .023 |  |  |
| Continuity Correction^b^ | 4.817 | 1 | .028 |  |  |
| Likelihood Ratio | 5.055 | 1 | .025 |  |  |
| Fisher's Exact Test |  |  |  | .028 | .015 |
| Linear-by-Linear Association | 5.164 | 1 | .023 |  |  |
| N of Valid Cases | 1187 |  |  |  |  |
| a. 0 cells (0.0%) have expected count less than 5. The minimum expected count is 74.53. | | | | | |
| b. Computed only for a 2x2 table | | | | | |

**Tumorstage * Galectin3status**

| **Crosstab** | | | | |
| --- | --- | --- | --- | --- |
| Count | | | | |
|  | | Galectin3status | | Total |
|  |  | Negative | Positive |  |
| Tumorstage | DCIS | 154 | 97 | 251 |
|  | IDC | 645 | 291 | 936 |
| Total | | 799 | 388 | 1187 |

| **Chi-Square Tests** | | | | | |
| --- | --- | --- | --- | --- | --- |
|  | Value | df | Asymptotic Significance (2-sided) | Exact Sig. (2-sided) | Exact Sig. (1-sided) |
| Pearson Chi-Square | 5.135^a^ | 1 | .023 |  |  |
| Continuity Correction^b^ | 4.798 | 1 | .028 |  |  |
| Likelihood Ratio | 5.034 | 1 | .025 |  |  |
| Fisher's Exact Test |  |  |  | .028 | .015 |
| Linear-by-Linear Association | 5.131 | 1 | .024 |  |  |
| N of Valid Cases | 1187 |  |  |  |  |
| a. 0 cells (0.0%) have expected count less than 5. The minimum expected count is 82.05. | | | | | |
| b. Computed only for a 2x2 table | | | | | |

**Histologicalgrade * Galectin3status**

| **Crosstab** | | | | |
| --- | --- | --- | --- | --- |
| Count | | | | |
|  | | Galectin3status | | Total |
|  |  | Negative | Positive |  |
| Histologicalgrade | I | 72 | 43 | 115 |
|  | II | 694 | 78 | 772 |
|  | III | 33 | 267 | 300 |
| Total | | 799 | 388 | 1187 |

| **Chi-Square Tests** | | | |
| --- | --- | --- | --- |
|  | Value | df | Asymptotic Significance (2-sided) |
| Pearson Chi-Square | 612.477^a^ | 2 | .000 |
| Likelihood Ratio | 634.858 | 2 | .000 |
| Linear-by-Linear Association | 314.461 | 1 | .000 |
| N of Valid Cases | 1187 |  |  |
| a. 0 cells (0.0%) have expected count less than 5. The minimum expected count is 37.59. | | | |

**Tumorsize * Galectin3status**

| **Crosstab** | | | | |
| --- | --- | --- | --- | --- |
| Count | | | | |
|  | | Galectin3status | | Total |
|  |  | Negative | Positive |  |
| Tumorsize | T1 | 156 | 47 | 203 |
|  | T2 | 557 | 318 | 875 |
|  | T3 | 76 | 17 | 93 |
|  | T4 | 10 | 6 | 16 |
| Total | | 799 | 388 | 1187 |

| **Chi-Square Tests** | | | |
| --- | --- | --- | --- |
|  | Value | df | Asymptotic Significance (2-sided) |
| Pearson Chi-Square | 22.644^a^ | 3 | .000 |
| Likelihood Ratio | 24.018 | 3 | .000 |
| Linear-by-Linear Association | .719 | 1 | .396 |
| N of Valid Cases | 1187 |  |  |
| a. 0 cells (0.0%) have expected count less than 5. The minimum expected count is 5.23. | | | |

**Metastaticnodes * Galectin3status**

| **Crosstab** | | | | |
| --- | --- | --- | --- | --- |
| Count | | | | |
|  | | Galectin3status | | Total |
|  |  | Negative | Positive |  |
| Metastaticnodes | Negative | 443 | 175 | 618 |
|  | Positive | 356 | 213 | 569 |
| Total | | 799 | 388 | 1187 |

| **Chi-Square Tests** | | | | | |
| --- | --- | --- | --- | --- | --- |
|  | Value | df | Asymptotic Significance (2-sided) | Exact Sig. (2-sided) | Exact Sig. (1-sided) |
| Pearson Chi-Square | 11.191^a^ | 1 | .001 |  |  |
| Continuity Correction^b^ | 10.781 | 1 | .001 |  |  |
| Likelihood Ratio | 11.196 | 1 | .001 |  |  |
| Fisher's Exact Test |  |  |  | .001 | .001 |
| Linear-by-Linear Association | 11.182 | 1 | .001 |  |  |
| N of Valid Cases | 1187 |  |  |  |  |
| a. 0 cells (0.0%) have expected count less than 5. The minimum expected count is 185.99. | | | | | |
| b. Computed only for a 2x2 table | | | | | |

**Her2status * Galectin3status**

| **Crosstab** | | | | |
| --- | --- | --- | --- | --- |
| Count | | | | |
|  | | Galectin3status | | Total |
|  |  | Negative | Positive |  |
| Her2status | Negative | 574 | 291 | 865 |
|  | Positive | 225 | 97 | 322 |
| Total | | 799 | 388 | 1187 |

| **Chi-Square Tests** | | | | | |
| --- | --- | --- | --- | --- | --- |
|  | Value | df | Asymptotic Significance (2-sided) | Exact Sig. (2-sided) | Exact Sig. (1-sided) |
| Pearson Chi-Square | 1.319^a^ | 1 | .251 |  |  |
| Continuity Correction^b^ | 1.164 | 1 | .281 |  |  |
| Likelihood Ratio | 1.331 | 1 | .249 |  |  |
| Fisher's Exact Test |  |  |  | .266 | .140 |
| Linear-by-Linear Association | 1.318 | 1 | .251 |  |  |
| N of Valid Cases | 1187 |  |  |  |  |
| a. 0 cells (0.0%) have expected count less than 5. The minimum expected count is 105.25. | | | | | |
| b. Computed only for a 2x2 table | | | | | |

**Triplenegativebreastcancer * Galectin3status**

| **Crosstab** | | | | |
| --- | --- | --- | --- | --- |
| Count | | | | |
|  | | Galectin3status | | Total |
|  |  | Negative | Positive |  |
| Triplenegativebreastcancer | No | 704 | 257 | 961 |
|  | Yes | 95 | 131 | 226 |
| Total | | 799 | 388 | 1187 |

| **Chi-Square Tests** | | | | | |
| --- | --- | --- | --- | --- | --- |
|  | Value | df | Asymptotic Significance (2-sided) | Exact Sig. (2-sided) | Exact Sig. (1-sided) |
| Pearson Chi-Square | 81.062^a^ | 1 | .000 |  |  |
| Continuity Correction^b^ | 79.649 | 1 | .000 |  |  |
| Likelihood Ratio | 76.612 | 1 | .000 |  |  |
| Fisher's Exact Test |  |  |  | .000 | .000 |
| Linear-by-Linear Association | 80.993 | 1 | .000 |  |  |
| N of Valid Cases | 1187 |  |  |  |  |
| a. 0 cells (0.0%) have expected count less than 5. The minimum expected count is 73.87. | | | | | |
| b. Computed only for a 2x2 table | | | | | |
