# Supplementary material for: Correction: Galectin-3 as a Marker and Potential Therapeutic Target in Breast Cancer
Source: PLoS One. 2020 Apr 16;15(4):e0232166. doi: 10.1371/journal.pone.0232166 (PMC7162513; doi:10.1371/journal.pone.0232166)
Supplement: S13 File — (DOCX) [file pone.0232166.s013.docx]

**Cox Regression**

| **Case Processing Summary** | | | |
| --- | --- | --- | --- |
|  | | N | Percent |
| Cases available in analysis | Event^a^ | 431 | 36.3% |
|  | Censored | 756 | 63.7% |
|  | Total | 1187 | 100.0% |
| Cases dropped | Cases with missing values | 0 | 0.0% |
|  | Cases with negative time | 0 | 0.0% |
|  | Censored cases before the earliest event in a stratum | 0 | 0.0% |
|  | Total | 0 | 0.0% |
| Total | | 1187 | 100.0% |
| a. Dependent Variable: DFSmonth | | | |

**Block 0: Beginning Block**

| **Omnibus Tests of Model Coefficients** |
| --- |
| -2 Log Likelihood |
| 5481.948 |

**Block 1: Method = Enter**

| **Omnibus Tests of Model Coefficients^a^** | | | | | | | | | |
| --- | --- | --- | --- | --- | --- | --- | --- | --- | --- |
| -2 Log Likelihood | Overall (score) | | | Change From Previous Step | | | Change From Previous Block | | |
|  | Chi-square | df | Sig. | Chi-square | df | Sig. | Chi-square | df | Sig. |
| 4964.992 | 744.104 | 8 | .000 | 516.956 | 8 | .000 | 516.956 | 8 | .000 |
| a. Beginning Block Number 1. Method = Enter | | | | | | | | | |

| **Variables in the Equation** | | | | | | | | |
| --- | --- | --- | --- | --- | --- | --- | --- | --- |
|  | B | SE | Wald | df | Sig. | Exp(B) | 95.0% CI for Exp(B) | |
|  |  |  |  |  |  |  | Lower | Upper |
| Age | -2.243 | .497 | 20.336 | 1 | .000 | .106 | .040 | .281 |
| Tumorsize | .940 | .123 | 58.051 | 1 | .000 | 2.561 | 2.011 | 3.262 |
| Histologicalgrade | .662 | .182 | 13.216 | 1 | .000 | 1.939 | 1.357 | 2.771 |
| Tumorstage | 1.212 | .418 | 8.426 | 1 | .004 | 3.361 | 1.483 | 7.621 |
| Metastaticnodes | 1.513 | .131 | 133.002 | 1 | .000 | 4.539 | 3.510 | 5.869 |
| Her2status | .273 | .240 | 1.291 | 1 | .256 | 1.314 | .821 | 2.104 |
| Galectin3status | .809 | .164 | 24.245 | 1 | .000 | 2.246 | 1.627 | 3.099 |
| Triplenegativebreastcancer | -.162 | .165 | .958 | 1 | .328 | .851 | .615 | 1.176 |

| **Covariate Means** | |
| --- | --- |
|  | Mean |
| Age | .808 |
| Tumorsize | 1.934 |
| Histologicalgrade | 2.156 |
| Tumorstage | .789 |
| Metastaticnodes | .479 |
| Her2status | .271 |
| Galectin3status | .327 |
| Triplenegativebreastcancer | .190 |
